# Melatonin Induces PERK‐ATF4 Unfolded Protein Response and Apoptosis in Human Choriocarcinoma Cells

**DOI:** 10.1111/jpi.70072

**Published:** 2025-08-27

**Authors:** Josianne Bienvenue‐Pariseault, Lucas Sagrillo‐Fagundes, Philippe Wong‐Yen, Darius Stakamatos, Marie Cohen, Cathy Vaillancourt

**Affiliations:** ^1^ Institut national de la recherche scientifique (INRS) ‐ Centre Armand‐Frappier Santé Biotechnologie (AFSB) Laval Canada; ^2^ CIUSSS‐Nord‐de‐l'île‐de‐Montréal Montreal Canada; ^3^ Department of Pediatrics, Gynecology and Obstetrics, Faculty of Medicine and Translational Research Centre in Onco‐hematology University of Geneva Geneva Switzerland

**Keywords:** apoptosis, BeWo cell, choriocarcinoma, indolamine, melatonin, PERK pathway, unfolded protein response

## Abstract

Melatonin, an indolamine primarily recognized for regulating circadian rhythms, has also demonstrated notable antitumoral properties. Melatonin induces endoplasmic reticulum (ER) stress, modulates autophagy, and promotes apoptosis in various tumors, including gastric, ovarian, cervical, oral tongue, colorectal, renal, hepatic, and bladder cancer. In placental choriocarcinoma, melatonin reduces cell viability and induces apoptosis by inhibiting autophagy and disrupting the mitochondrial membrane potential. However, its effects on ER stress and the unfolded protein response (UPR) pathway remain unexplored. It is hypothesized here that the proapoptotic effects of melatonin in choriocarcinoma cells occur through the activation of the UPR pathway. The factors implicated in the UPR (PERK, IRE1ɑ, ATF6, GRP78, ATF4, CHOP, P‐eIF2α) pathways were evaluated by Western blot, RT‐qPCR, and flow cytometry in BeWo (human choriocarcinoma) cells treated with or without melatonin (1 mM). Melatonin significantly increased protein levels of GRP78 (*p* = 0.0329), IRE1α (*p* = 0.0394), p‐eIF2α (*p* = 0.0439), ATF4 (*p* = 0.0267), CHOP (*p* = 0.0379), Bax and cleaved PARP but did not affect TRAF2 and NFkB protein levels nor XBP1 mRNA splicing. PERK knockdown, via siRNA, prevented the rise in GRP78, p‐eIF2α/eIF2α, and ATF4 levels by melatonin. Additionally, melatonin increased early apoptosis in BeWo cells (*p* = 0.0371) and PERK knockdown increased the susceptibility of BeWo cells to apoptosis when treated with tunicamycin (*p* = 0.0359), suggesting that ER stress plays a role in BeWo cell survival. This study demonstrates that melatonin activates the PERK‐ATF4‐P‐eIF2α‐CHOP pathway and induces early apoptosis in BeWo cells, while PERK deficiency compromises cell survival under ER stress. Our findings suggest that modulating PERK‐UPR signaling with melatonin could present a promising therapeutic strategy for cancer, including placental choriocarcinoma.

## 1 Introduction

The endoplasmic reticulum (ER) is a multitask organelle that assures Ca^2+^ storage/distribution, lipid biosynthesis, translation, posttranslational modifications and folding of proteins [1]. Physiological stressors, such as nutrient deprivation [2], acidosis [3], and oxidative stress [4], can compromise ER functions, thereby inducing ER stress. Altered ER function can lead to an accumulation of unfolded or misfolded proteins that trigger the unfolded protein response (UPR). The UPR can restore cell homeostasis by expressing chaperones that help in folding proteins. This process eliminates unfolded proteins through autophagy and proteasomal degradation, primarily via the activation of protein kinase RNA‐like endoplasmic reticulum kinase (PERK), inositol‐requiring transmembrane kinase/endonuclease 1α (IRE1α) and activating transcription factor 6 (ATF6) [5, 6, 7]. When the ER stress is prolonged, the UPR pathway can no longer restore cellular homeostasis, shifting instead to a proapoptotic response and away from an adaptive response [8, 9].

The UPR pathway has significant roles in tumorigenesis, metastasis, and survival. PERK, IRE1α and ATF6 are crucial for the adaptation of cancer cells to their changing environment [10]. Anticancer drugs, with reactive oxygen species (ROS)‐modulating ability, have been shown to counteract cancer cell adaptation by inducing ROS generation causing excessive ER stress, thereby promoting apoptosis in cancer cells [11]. For example, many studies showed that *N*‐acetyl‐5‐methoxytryptamine (melatonin) induces apoptosis through the UPR pathway activation in gastric [12], bladder [13], hepatic [14, 15], skin [16], tongue squamous carcinoma [17], and colorectal human cancer [18].

Melatonin is widely known as pineal gland derived circadian regulator [19]. However, this amphiphilic indolamine is also synthesized by all organs and tissues, including placental trophoblast cells [20, 21]. Melatonin can cross easily through lipophilic barriers and is known to have anti‐inflammatory [22], antioxidants [23], antiapoptotic [24], and antitumoral functions [25] in healthy cells subjected to stressful conditions. Paradoxally, melatonin has been shown to inhibit cell proliferation [26], migration [27], angiogenesis [28], and metastasis [25], as well as inducing metabolic dysregulation [29], promoting apoptosis [30], and triggering pro‐inflammatory [31] responses across diverse tumor cell types [32, 33]. The use of melatonin as a therapeutic agent in cancer is promising due to its low toxicity, ease of synthesize and its ability to protect healthy cells submitted to stress, including chemotherapy side‐effects [34, 35, 36, 37]. Indeed, we have previously shown that melatonin induces choriocarcinoma cell death by inhibiting autophagy, decreasing cell viability and disrupting mitochondrial membrane potential whilst, in contrast, being protective in normal primary human villous trophoblast submitted to hypoxia/reoxygenation (H/R) by inducing autophagy, reducing oxidative stress and inflammation [35, 38, 39, 40]. However, the effect of melatonin on ER stress in choriocarcinoma cells has never been studied. In tongue squamous carcinoma [17], colorectal carcinoma [41], lung [42], liver [15, 43] and cervical cancer [44], induction of oxidative stress and autophagy by melatonin was associated with the induction of ER stress [33].

Choriocarcinoma is a rare and highly aggressive neoplasm that originates from the malignant transformation of trophoblasts [45]. Even though most choriocarcinoma patients are successfully treated with a single‐agent chemotherapy, others die despite multi‐drug resistance [46]. Therefore, novel therapy strategies are needed to improve the prognosis of this group of patients [46]. Utilizing melatonin to disrupt UPR signaling and alter the delicate equilibrium between adaptive UPR and UPR‐induced apoptosis could present an innovative therapeutic strategy [47, 48].

Overall, we propose that melatonin induces UPR pathways in choriocarcinoma cells. Therefore, the present study aimed to investigate melatonin antitumoral effects through the modulation of UPR in BeWo choriocarcinoma cells.

## 2 Materials and Methods

### 2.1 BeWo Cell Line Culture

BeWo cells (#CCL‐98; ATCC, MD, US) were cultured in T‐75 flask cm^2^ (Corning Life Science, Arizona, US) at 37°C and 5% CO_2_, as previously described [38]. The culture medium used was Dulbecco's modified Eagle's medium/Ham's F‐12 nutrient mixture (DMEM/F12) without phenol red, supplemented with 0.6 g/L sodium bicarbonate (NaHCO₃) (Sigma‐Aldrich, ON, Canada) and 10% fetal bovine serum (FBS; Wisent, QC, Canada), and was refreshed every 2 days. Subculturing was performed when cells reached 90%–95% of confluence.

### 2.2 Cell Treatments

Cells between passages 6 and 18 were seeded at a density of 2.5 × 105 cells/mL in 6‐well plate and cultured under physiological normoxia (8% O_2_, 5% CO_2_, 87% N_2_) for 24‐h, in a Modular Incubator Chambers (Billups‐Rothenberg, CA, USA), to ensure cell adherence, as we previously described [49]. Subsequently, the cells were treated with melatonin (Mel, 1 mM) (Sigma‐Aldrich) or with the vehicle control dimethyl sulfoxide (DMSO; Sigma‐Aldrich) at 0.1% (final concentration) or with 3.5 μg/mL of tunicamycin (Tm, ER stress positive control; Cayman chemicals, MI, US) and maintained under normoxia for 24‐h. Melatonin solution diluted in 0.1% DMSO was freshly prepared before each experiment [38]. A dose–response curve was conducted using increasing melatonin concentrations (1 nM, 1 µM, and 1 mM) to determine the concentration with the maximal effect, with melatonin (1 mM) being selected (Figure S1).

### 2.3 Protein Expression Analysis

Protein expression of BeWo cells treated with or without melatonin was analyzed by immunoblot [22, 38, 50]. Proteins were extracted using RIPA buffer (50 mM TRIS‐HCl pH 7.4, 1 mM EDTA, 150 mM NaCl, NP‐40 (Nonidet P‐40) 1%, Na‐deoxycholate 0.25%) supplemented with protease (Invitrogen, ON, Canada) and phosphatase inhibitors (Roche, ON, Canada). The concentration in each sample was determined by spectrometry using the bicinchoninic acid (BCA) assay according to the manufacturer's instructions (Pierce, Biotechnology, CA, USA). Proteins (35 µg) were separated by electrophoresis on 8%, 12% or 10%–15% SDS‐PAGE and transferred to nitrocellulose membranes using the Trans‐Blot Turbo (BioRad, QC, Canada). To block nonspecific binding, the membrane was incubated in Tris‐buffered saline (TBS) containing 5% bovine serum albumin (BSA) or 5% nonfat dry milk. Primary antibodies were incubated overnight at 4°C (Table 1). Subsequently, incubation with the appropriate HRP‐conjugated secondary antibodies was carried out for 1‐h at room temperature (Table 1). Blots were developed with Clarity Western ECL substrate or Clarity Max Western ECL substrate (BioRad) and the chemiluminescence was detected with the ChemiDoc XRS+ system (BioRad). Band quantification was carried out by densitometric analysis, using Image Lab 5.2 software (BioRad). The normalization of the proteins was done with total protein using the MemCode Reversible Stain Kit, according to the manufacturer's instructions (Thermo Fisher Scientific, ON, Canada). Total protein normalization was used to avoid variability under stress conditions and ensure accurate quantification as previously described [50, 51]. Before this, we prepared a standard curve using serial dilutions of the protein samples to determine the optimal concentration of primary antibodies to use according to Taylor et al. recommendations [50, 51].

**Table 1:** List of antibodies used for Western blot analysis.

Antibody	Source	Molecular weight (kDa)	Dillution	Supplier
GRP78	Rabbit	78	1:1000	Cell signaling, #3177
IRE1α	Rabbit	130	1:1000	Cell signaling, #3294
TRAF2	Rabbit	53	1:1000	Abcam, #ab126758
PERK	Rabbit	140	1:1000	Cell signaling, #5683
Phospho‐eIF2 α	Rabbit	38	1:500	Abcam, #32157
eIF2 α	Rabbit	38	1:500	Cell signaling, #9722
ATF4	Rabbit	49	1:500	Cell signaling, #11815
CHOP	Rabbit	27	1:1000	Cell sgnaling, #2895
NF‐κB (p65)	Rabbit	65	1:1000	Cell signaling, #8242
IκB	Rabbit	39	1:1000	Cell signaling, #4812
Bax	Rabbit	20	1:500	Cell signaling, #2774
Bcl‐2	Mouse	26	1:500	Cell signaling, #15071
ATF6ɑ	Rabbit	74	1:500	Abcam, #ab227830
Cl‐PARP	Rabbit	89	1:500	Cell Signaling, #5625
Anti‐Mouse HRP	—	—	1:5000	Millipore (AP192P)
Anti‐Rabbit HRP	—	—	1:5000	Millipore (AP182P)

Abbreviations: ATF4, activating transcription factor 4; Bax, Bcl‐2–associated X; Bcl‐2, B‐cell lymphoma 2; CHOP, DNA damage‐inducible transcript 3, also known as C/EBP homologous protein; eif2α, eukaryotic initiation factor 2; GRP78, 78‐kDa glucose‐regulated protein; HRP, horseradish peroxidase; IkB, inhibitor of nuclear factor kappa B; IREα, serine/threonine‐protein kinase/endoribonuclease inositol‐requiring enzyme 1 α; NFkB, nuclear factor‐kappa; P‐eif2α, phosphorylation of the eukaryotic initiation factor 2; TRAF2, TNF receptor‐associated factor 2; XBP1, X‐box binding protein 1.

### 2.4 mRNA Expression Analysis

BeWo cells treated or not with melatonin were analyzed by real time quantitative polymerase chain (RT‐qPCR), according to the MIQE guidelines [52, 53]. Total RNA was extracted with the RNeasy mini kit, according to the manufacturer's instructions (Qiagen, ON, Canada). RNA purity and integrity were respectively assessed by spectrometry with the Nanodrop and by electrophoresis of the RNA on agarose gel, respectively. The samples that satisfied the purity and the integrity were processed to cDNA using the iScript Reverse Transcription Supermix for RT‐qPCR (BioRad). cDNA was then amplified with the SsoAdvanced Universal SYBR Green Supermix (BioRad) and specific primers (Table 2). Beforehand, amplification efficacity of the primers was determined by doing a standard curve and primer's annealing temperature was confirmed by doing a thermal gradient. Experiments were run on a CFX96 Real‐Time PCR Detection System (BioRad). Gene expression was normalized to beta‐2‐microglobubin (B2M) and succinate dehydrogenase complex flavoprotein subunit A (SDHA) and expressed as a relative expression.

**Table 2:** Primers used for RT‐qPCR analysis.

Primers	Primer sequences (5′ – > 3′)	Amplicon size (nt)	References
**XBP1s**	Foward: TGCTGAGTCCGCAGCAGGTG Reverse: GCTGGCAGGCTCTGGGGAAG	169	(von Loeffelholz et al., 2017)
**XBP1 total**	Foward: GTGAGCTGGAACAGCAAGTGGT Reverse: CCAAGCGCTGTCTTAACTCCTG	126	(von Loeffelholz et al., 2017)
**B2M**	Foward: GATGAGTATGCCTGCCGTGT Reverse: CTGCTTACATGTCTCGATCCCA	79	Primer Blast
**SDHA**	Foward: TACAAGGTGCGGATTGATG Reverse: CGATCACGGGTCTATATTCAA	148	Primer Blast

Abbreviations: B2M, beta‐2‐microglobubin; SDHA, succinate dehydrogenase complex flavoprotein subunit A; XBP1s, cleaved X‐box binding protein; XBP1, X‐box binding protein 1.

### 2.5 Quantification of Apoptosis by Flow Cytometry

Annexin V‐(Fluorescein) FITC/Propidium iodide (PI) kit (eBiosciences, ON, Canada) was used to analyze early and late apoptosis by flow cytometry. As a positive control, BeWo cells were treated with staurosporine (1 mM) (Sigma‐Aldrich) for 24‐h. Cells were harvested with TrypLE Express Enzyme 1× (Gibco, ON, Canada) and washed with PBS. Cells (2.5 × 105 cells/mL) were resuspended in binding buffer provided by the manufacturer. Annexin V‐FITC and PI staining were done according to the manufacturer's instructions. Briefly, 5 μL of Annexin V‐FITC was added to 195 μL of cell suspension and incubated for 10‐min at room temperature. PI (20 μg/mL) was added to one sample at a time, for 1‐min at room temperature just before reading, to decrease the false positive risk. Quantification of apoptotic cells by flow cytometry was done using FACSCalibur (BD Biosciences, CA, US). Data were analyzed by using FlowJo software (BD Biosciences).

### 2.6 siRNA Reverse Transfection

Human PERK‐specific short interfering RNA (siRNA) or scrambled RNA (control) was mixed with serum‐free Opti‐MEM medium for 5‐min before adding Lipofectamine RNAiMAX for 20‐min in a 24‐well plate (Life Technologies, CA, USA). BeWo cells (17.5 × 10⁴ cells/mL) were then added to the wells and cultured for 72‐h under normoxic conditions. Subsequently, cells were treated with DMSO (vehicle control), melatonin, or tunicamycin for 24‐h in normoxia. Human PERK‐specific siRNA and scrambled RNA were purchased from Santa Cruz (TX, US).

### 2.7 Statistical Analysis

All data represent at least four different BeWo cell passages. Statistically significant differences (*p* < 0.05) were assessed using GraphPad Prism 9 (version 9.5.1). Student *t*‐test was used when two treatments groups were compared while one‐way analysis of variance (ANOVA) followed by the Tukey post‐hoc test was used when more than two treatment groups were compared.

## 3 Results

### 3.1 Melatonin Activated the Unfolded Protein Response (UPR) in BeWo Cells

**Figure 1:**
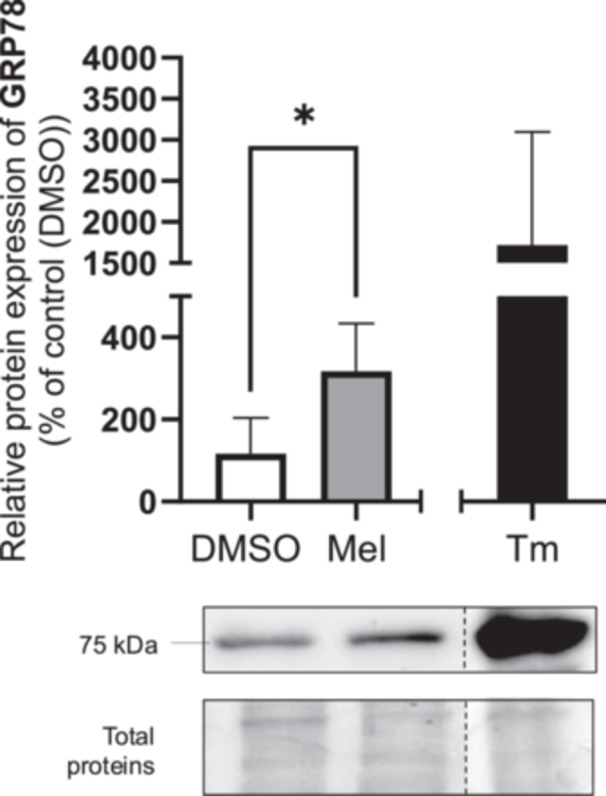
Melatonin increases unfolded protein (UPR) response in BeWo cells. BeWo cells were treated with the vehicle control (0.1% DMSO) with or without melatonin (1 mM) or Tm (3.5 µg/mL) under normoxia (8% O_2_) conditions for 24‐h. GRP78 protein level was determined by Western blot. Equal protein amounts of cell lysates (35 µg) were subjected to Western blot assay using anti‐GRP78. Total protein was used for normalization. GRP78: 78‐kDa glucose‐regulated protein; DMSO: dimethylsulfoxyde; Mel: melatonin; Tm: municamycin; Data are shown as mean ± SD and were analyzed using an unpaired *t*‐test (DMSO vs Mel, *p < 0.05), *n* = 5. To facilitate readers' experiences, the certain band order has been changed. Those modifications have been identified with dotted lines. Original western blots are included under Figure S2.

To determine if melatonin activates the UPR in BeWo cells, we investigated the protein levels of the first UPR mediators, namely Glucose‐Regulated Protein 78 (GRP78). Tunicamycin, used as positive control for UPR activation, increased GRP78 protein level compared to vehicle control. However, as a positive control, tunicamycin was not included in the statistical analysis. Melatonin (1 mM) increased GRP78 protein level by 3.2‐fold in BeWo cells compared to the vehicle control (Figure 1). This suggests that melatonin activate the UPR in BeWo cells.

### 3.2 Melatonin Has No Effect on the ER Membrane Transducer IREα and ATF6 Pathways

**Figure 2:**
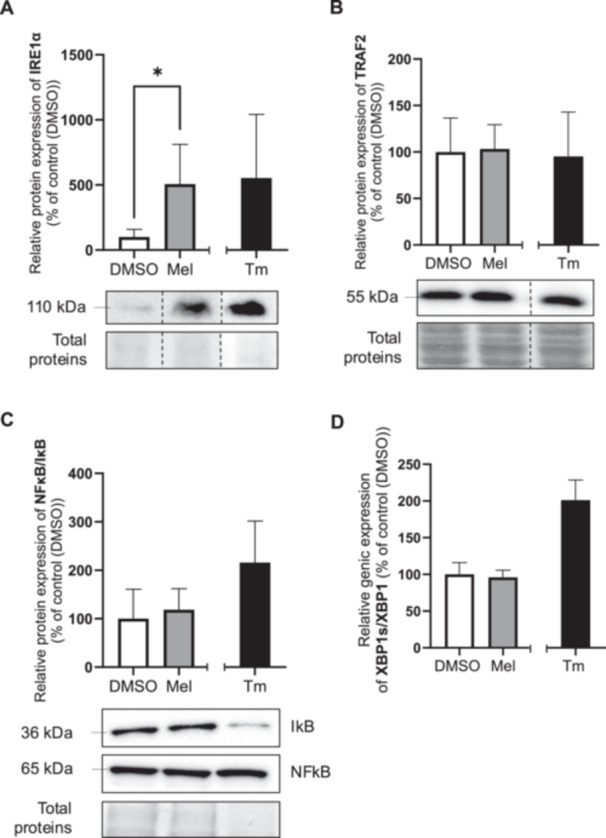
Melatonin has no effect on the protein level of factors implicated in IREα‐UPR pathway. BeWo cells were treated with the vehicle (DMSO 0.1%) or melatonin (1 mM) or Tm (3.5 μg/mL) under normoxia (8% O_2_) conditions during 24‐h. (A) IREα, (B) TRAF2 and (C) IkB/NFkB ratio protein level was determined by Western blot. Equal protein amounts of cell lysates (35 µg) were subjected to Western blot assay using anti‐IREα, anti‐TRAF2, anti‐NFkB and anti‐IkB. Total protein was used for normalization. (D) XBP1s/XBP1 gene expression was determined by RT‐qPCR using specific primers. *B2M* and *SDHA* were used as references genes for normalization. DMSO: dimethylsulfoxyde; IkB: inhibitor of nuclear factor kappa B; IREα: serine/threonine‐protein kinase/endoribonuclease inositol‐requiring enzyme 1α; Mel: melatonin; NFkB: nuclear factor‐kappa B; TRAF2: TNF receptor‐associated factor 2; Tm: Tunicamycin; XBP1: X‐box binding protein 1; XBP1s: spliced X‐box binding protein 1; Data are shown as mean ± SD and were analyzed using an unpaired t‐test (DMSO vs Mel, *p < 0.05), *n* = 4–5. To facilitate readers' experiences, the band order of certain blots has been changed. Those modifications have been identified with dotted lines. Original western blots are included under Figures S5–S8.

**Figure 3:**
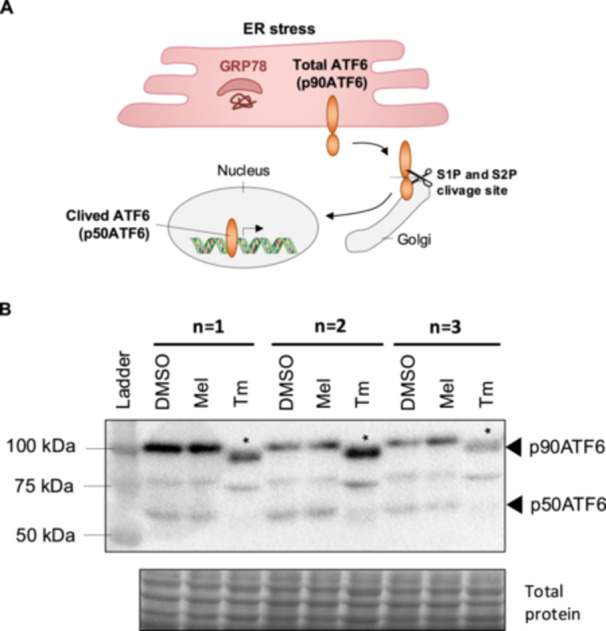
Melatonin has no effect on ATF6 protein cleavage. BeWo cells were treated with the vehicle (DMSO 0.1%) or melatonin (1 mM) or Tm (3.5 μg/mL) under normoxia (8% O_2_) conditions during 24‐h. (A) Following GRP78 dissociation, ATF6 (p90ATF6, total ATF6) translocates to the Golgi apparatus, where it undergoes proteolytic cleavage by site‐1 protease (S1P or MBTPS1) and site‐2 protease (S2P or MBTPS2). The resulting activated form, p50ATF6, then enters the nucleus to promote the transcription of genes containing ER stress‐response elements (ERSE‐1). (B) ATF6 protein cleavage level was determined by Western blot. Equal protein amounts of cell lysates were subjected to Western blot assay using anti‐ATF6 antibody. Total protein was used for normalization. ATF6, activating transcription factor 6; DMSO, dimethylsulfoxyde; ER, endoplasmic reticulum; GRP78, 78‐kDa glucose‐regulated protein; Mel, melatonin; S1P, site‐1 protease; S2P, site‐1 protease; Tm, tunicamycin.

To investigate whether melatonin activates the IRE1α and ATF6 arms of the UPR in BeWo cells, we assessed the expression of key proteins and genes involved in these pathways. Specifically, to evaluate the IRE1α arm, we measured the protein levels of TNF receptor‐associated factor 2 (TRAF2), Nuclear factor‐kappa B (NFκB), and inhibitor of nuclear factor kappa B (IκB) by immunoblotting [54, 55]. In addition, we analyzed the gene expression of *XBP1* (X‐box binding protein 1) and its spliced form, *sXBP1*, using RT‐qPCR (Figure 2). As with tunicamycin, melatonin significantly increased IRE1α protein level (5.1‐fold) compared to the vehicle control (Figure 2A). However, TRAF2 protein levels remained unchanged following exposure to melatonin or tunicamycin, compared to the vehicle control (Figure 2B). In addition, melatonin had no effect on the NFκB/IκB or *XBP1/XBP1s* ratios, whereas tunicamycin increased both (Figure 2C,D). Although tunicamycin was run as a positive control, tunicamycin was not included in the statistical analysis. The expression levels of the individual proteins or genes used to calculate the NFκB/IκB and *XBP1/XBP1s* ratios can be found in the Supporting data (Figure S3). A time course analysis (2, 4, 6, and 24‐h) confirmed that melatonin did not induce changes in TRAF2, IκB/NFκB, or *XBP1/XBP1s* expression at any of these time point (Figure S4). These results indicate that melatonin does not activate the downstream pathways of IRE1α in BeWo cells. To evaluate ATF6 arm, we assessed ATF6 proteolysis, qualitatively, by Western blot [56, 57] (Figure 3A). Tunicamycin, which induces ER stress by inhibiting protein glycosylation, produced a faster‐migrating band of total ATF6 (p90ATF6) compared to the vehicle control and melatonin. This suggests the formation of an unglycosylated ATF6 precursor (p90ATF6*) as reported by other studies [7, 58] (Figure 3B). Tunicamycin treatment led to a reduction in the cleaved form of ATF6 (p50ATF6), suggesting its nuclear translocation and activation—a process not observed with melatonin nor the vehicle control. This supports that tunicamycin activates ATF6, likely via proteolytic cleavage.

### 3.3 Melatonin Activates the Pathway of the ER Membrane Transducer Perk

**Figure 4:**
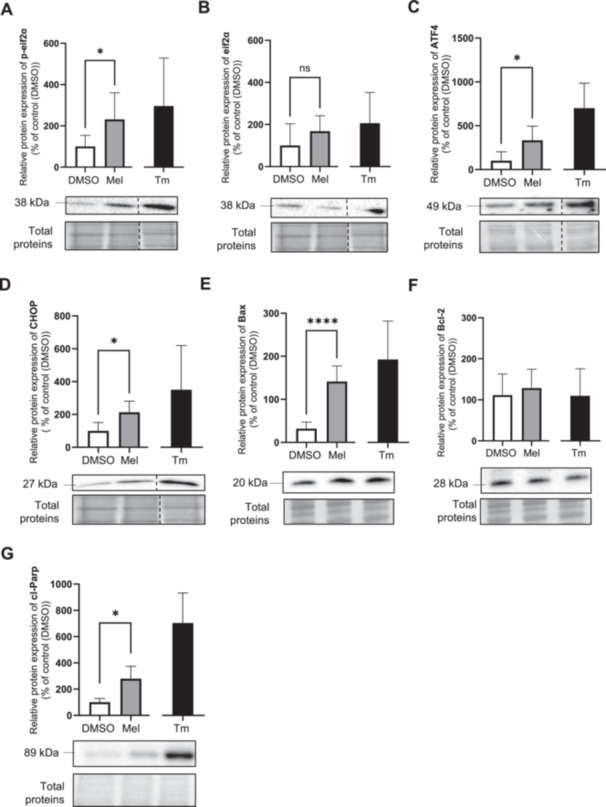
Melatonin increases the protein level of factors implicated in PERK‐UPR pathway. BeWo cells were treated with the vehicle (DMSO 0.1%) or melatonin (1 mM) or Tm (3.5 μg/mL) under normoxia (8% O_2_) conditions during 24‐h. (A) p‐eif2α, (B) eif2α, (C) ATF4, (D) CHOP, (E) Bax, (F) Bcl‐2, and (G) cl‐Parp protein level was determined by Western blot. Equal protein amounts of cell lysates (35 µg) were subjected to Western blot assay using anti‐p‐eif2α, anti‐eif2α, anti‐ATF4, anti‐CHOP, anti‐Bax, anti‐Bcl‐2 and anti‐cl‐Parp. Total protein was used for normalization. ATF4, activating transcription factor 4; Bax, Bcl‐2–associated X; Bcl‐2, B‐cell lymphoma 2; CHOP, DNA damage‐inducible transcript 3, also known as C/EBP homologous protein; cl‐Parp, cleaved poly (ADP‐ribose) polymerase; DMSO, dimethylsulfoxyde; eif2α, eukaryotic initiation factor 2 subunit alpha; Mel, melatonin; p‐eif2α, phosphorylation of the eukaryotic initiation factor 2 subunit alpha; Tm, tunicamycin. Data are shown as mean ± SD and were analyzed using an unpaired *t*‐test (DMSO vs. Mel, *p < 0.05), *n* = 4–6. To facilitate readers' experiences, the band order of certain blots has been changed. Those modifications have been identified with dotted lines. Original western blots are included under Figures S12–S18.

To determine whether melatonin activates the PERK arm of the UPR in BeWo cells, we assessed the protein levels of key downstream factors by Western blot, including Eukaryotic initiation factor 2 subunit alpha (eIF2α), phosphorylated p‐eIF2α (p‐eiF2α), Activating Transcription Factor 4 (ATF4), C/EBP homologous protein (CHOP), B‐cell lymphoma 2 (Bcl‐2), B‐cell lymphoma 2 associated X (Bax) and Cleaved poly (ADP‐ribose) polymerase (cl‐Parp) (Figure 4). As with the positive control tunicamycin, melatonin significantly increased (2.3‐fold) the protein level of p‐eIF2α compared to the vehicle control (Figure 4A), without affecting eIF2α levels (Figure 4B; see p‐eIF2α/eIF2α ratio in Figure S9). Melatonin significantly upregulated ATF4 (Figures 4C, 3.3‐fold), CHOP (Figures 4D, 2.1‐fold) and the proapoptotic protein Bax (Figures 4E, 1.4‐fold), while having no effect on the antiapoptotic protein Bcl‐2 (Figure 4F). Additionally, melatonin increased cl‐Parp protein levels (2.8‐fold) compared to vehicle control (Figure 4G). Tunicamycin, included as a positive control, was excluded from the statistical analysis. No effects of melatonin were observed PERK protein levels (Figure S10) or on ATF4 and CHOP gene expression (Figure S11). In summary, melatonin activates the PERK related UPR pathway by increasing p‐eiF2α, ATF4, cl‐Parp and Bax proteins levels in BeWo cells.

### 3.4 Melatonin Induces Early Apoptosis in BeWo Cells

**Figure 5:**
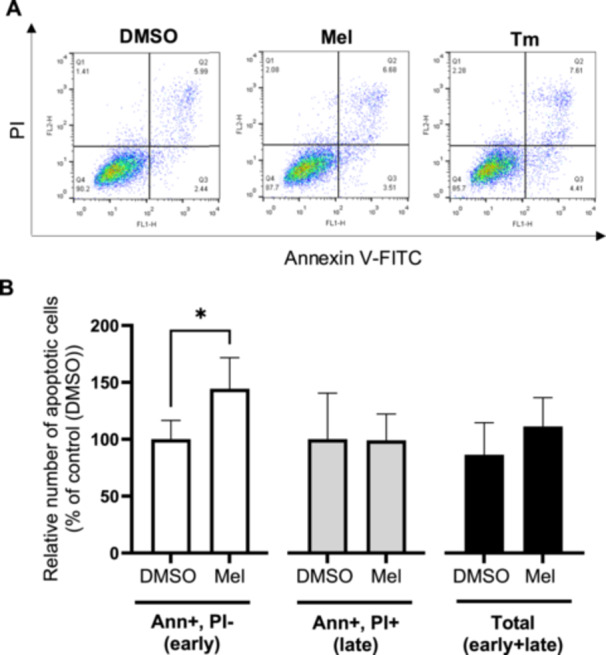
Melatonin increases early apoptosis in BeWo cells. BeWo cells were treated with the vehicle (DMSO) or melatonin (1 mM) or Tm (3.5 μg/mL) under normoxia (8% O_2_) conditions during 24‐h. (A) Number of apoptotic cells was determined by FACS using Annexin V and PI staining. (B) Number of early or late apoptotic cells are expressed as a percentage of the control DMSO. Ann, annexin V; DMSO, dimethylsulfoxyde; Early, early apoptosis; Late, late apoptosis; Mel, melatonin; PI, propidium iodide; Tm, tunicamycin; Total, total apoptosis. Data are shown as mean ± SD and were analyzed using an unpaired *t*‐test (DMSO vs. Mel, *p < 0.05), *n* = 7.

Instead of restoring homeostasis, UPR pathways trigger apoptosis under persistent ER stress conditions [59]. Melatonin has been shown to activate UPR pathways, leading to apoptosis in different tumor cells [12, 18, 60]. To investigate its proapoptotic effect in BeWo cells, we performed flow cytometry using Annexin V and PI staining (Figure 5A,B). Compared to the vehicle control, melatonin significantly increased the relative number of Annexin V‐positive cells (1.4‐fold), indicating activation of early apoptosis. However, melatonin did not affect the number of cells that were positive for both Annexin V and PI, suggesting no effect on late apoptosis. Staurosporine (St) was used as a positive control for apoptosis and confirmed the validity of our approach (Figure S19). These results indicate that melatonin specifically induces early apoptosis in BeWo cells.

### 3.5 PERK Knockdown by siRNA Inhibits the Effects of Melatonin on the UPR Signaling Pathways and Early Apoptosis

**Figure 6:**
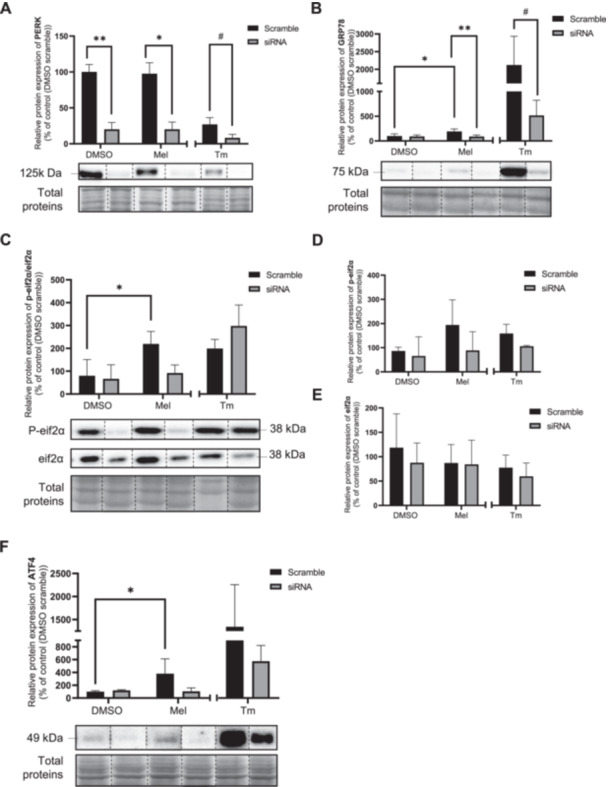
Knockdown of PERK inhibits the ability of melatonin to induce PERK‐UPR pathway. BeWo cells subjected to scramble (SC) or siRNA against PERK for 72‐h were treated with the vehicle (DMSO 0.1%) or melatonin (1 mM) or TM (3.5 μg/mL) under normoxia (8% O_2_) conditions during 24‐h. (A) PERK, (B) GRP78, (C) p‐eif2α/eif2α ratio (D) p‐eif2α, (E) eif2α, and (F) ATF4 protein level was determined by Western blot. Equal protein amounts of cell lysates (35 µg) were subjected to Western blot assay using anti‐PERK, anti‐GRP78, anti‐p‐eif2α, anti‐eif2α ratio and anti‐ATF4. Total protein was used for normalization. ATF4, activating transcription factor 4; DMSO, dimethylsulfoxyde; eif2α, eukaryotic initiation factor 2 subunit alpha; GRP78, 78‐kDa glucose‐regulated protein; Mel, melatonin; p‐eif2α, phosphorylation of the eukaryotic initiation factor 2 subunit alpha; PERK, protein kinase R (PKR)‐like; Tm, tunicamycin. Data are shown as mean ± SD and were analyzed using ANOVA followed by Tukey post hoc test (*p < 0.05), or an unpaired *t*‐test (SC Tm vs. si Tm, ^#^ *p* < 0.05, ^##^ *p* < 0.01), *n* = 5. To facilitate readers' experiences, the band order has been changed. Those modifications have been identified with dotted lines. Original western blots are included under Figures S22–S26.

**Figure 7:**
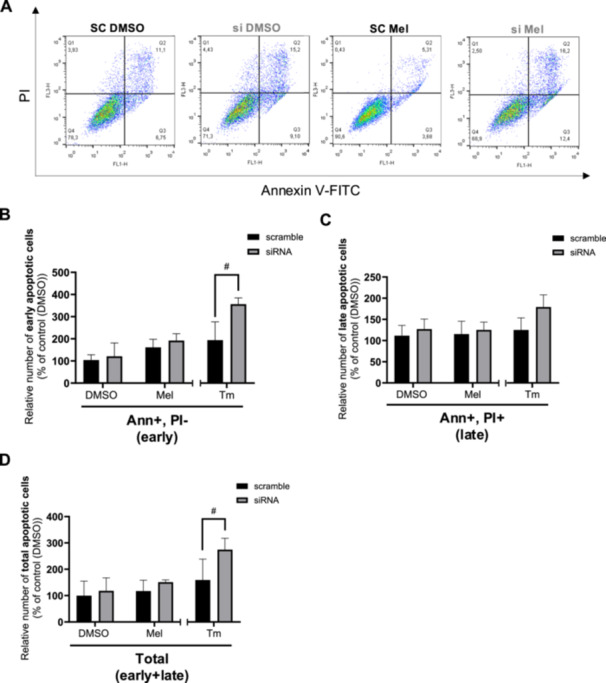
Melatonin and knockdown of PERK have no effect on early and late apoptosis. BeWo cells subjected to scramble (SC) or siRNA against PERK (si) for 72‐h were treated with the vehicle (DMSO 0.1%) or melatonin (1 mM) or Tm (3.5 μg/mL) under 8% of oxygen during 24‐h. (A) Apoptotic cells were determined by FACS using Annexin V and PI. Number of (B) early, (C) late or (D) total apoptotic cells are expressed as a percentage of the control DMSO. Ann, annexin V; DMSO, dimethylsulfoxyde; Early, early apoptosis; Late, late apoptosis; Mel, melatonin; PERK, (PKR)‐like protein kinase R; PI, propidium iodide; Tm, tunicamycin; Total, total (early + late) apoptosis. Data are shown as mean ± SD and were analyzed using ANOVA followed by Tukey post hoc test (*p < 0.05), or an unpaired *t*‐test (SC Tm vs si Tm, ^#^ *p* < 0.05, ^##^ *p* < 0.01). *n* = 5.

To determine whether melatonin's effects on the UPR are dependent on PERK, we performed PERK knockdown using siRNA. This approach was more efficient than the chemical inhibitor GSK2656157 (100 nM; 24‐h pretreatment), which failed to inhibit p‐eIF2α activation by tunicamycin (Figure S20). PERK knockdown was most effective 3 days posttransfection (Figure S21), leading to a 5.0‐fold decrease in PERK protein levels (Figure 6A). While melatonin increased GRP78 (Figure 6B), p‐eIF2α/eIF2α (Figure 6C,D,E), and ATF4 (Figure 6F) protein levels in scramble siRNA‐transfected cells, these effects were abolished in PERK‐knockdown cells. These findings suggest that PERK is essential for melatonin to exert its effects on the UPR pathway. To evaluate whether melatonin‐induced early apoptosis depends on PERK, we performed flow cytometry using Annexin V and PI staining on BeWo cells transfected with either scrambled siRNA or PERK siRNA (Figure 7A). Melatonin did not affect apoptosis in either condition (Figure 7B–D). Interestingly, PERK knockdown increased early (Figure 7B, 1.7‐fold) and total apoptosis (Figure 7D, 1.3‐fold) in tunicamycin‐treated cells compared to scramble siRNA, suggesting a role for PERK in BeWo cell survival.

## 4 Discussion

We have previously demonstrated that melatonin induces cell death in human BeWo choriocarcinoma cells line by inhibiting autophagy, disrupting mitochondrial membrane potential, and decreasing cell viability [35, 38, 40]. Additionally, studies have highlighted the interplay between cell death, the UPR pathway and melatonin in cancer [48, 61]. In this study, we explored melatonin's effect on the UPR in BeWo cells and found that it specifically activates PERK pathway, with no observable effect on the IRE1α or ATF6 arms. Consistent with this finding, melatonin treatment also increased GRP78 protein levels, suggesting the initiation of ER stress. Similar findings have been reported in gastric, colorectal, tongue, neck, and head cancers, where melatonin induces the PERK‐p‐eIF2α‐ATF4 axis [17, 18, 41, 62, 63].

Melatonin treatment increased IRE1α protein expression in BeWo cells. However, it did not affect *XBP1s/XBP1* gene expression or the protein levels of TRAF2 and NFκB/IκB, suggesting that melatonin does not activate this arm of the UPR. Once activated, IRE1α promotes the splicing of XBP1 mRNA, which supports the ER‐associated degradation (ERAD) pathway. In addition, IRE1α can trigger inflammatory signaling by recruiting TRAF2, which activates the IκB/NF‐κB pathway [54, 55]. In other cancers where melatonin activates the PERK axis, increased expression of IRE1α or p‐IRE1α expression has also been observed; however, TRAF2 or XBP1s expression were not assessed [17, 18, 41, 62].

We found that melatonin had no effect on ATF6 in BeWo cells, while other studies reported conflicting results. Some studies suggest inhibitory effects of melatonin on ATF6 cleavage while others indicate elevated ATF6 cleavage in different cancer cells [13, 17, 64]. Our data suggest that PERK is the predominant pathway activated by melatonin in BeWo cells, leading to persistent ER stress and the final stage of the UPR. Under severe or prolonged ER stress, the activity of IRE1α and ATF6 are attenuated, while the protective activity of PERK shift to a proapoptotic role via CHOP, ultimately leading to cell death [65, 66]. ATF6 and PERK are both activated upon GRP78 dissociation. ATF6 is cleaved in the Golgi to drive transcription of ER stress genes. PERK activation can lead to either phosphorylation of eIF2α, triggering ATF4‐dependent proapoptotic signaling, or activation of the Nuclear factor erythroid 2 related factor 2 (NRF2), which promotes cell survival—depending on the severity and duration of ER stress [54, 67]. Melatonin activation of the p‐eIF2α‐ATF4‐CHOP proapoptotic pathway suggests aa shift away from NRF2 and autophagy‐mediated pro‐survival response. This aligns with our previous findings, which demonstrated that melatonin inhibits NRF2 and autophagy while increasing proapoptotic factors (Bax, cl‐PARP) and reducing antiapoptotic factors (Bcl‐2) in BeWo cells [38]. Moreover, PERK knockdown prevented melatonin from activating GRP78, p‐eIF2α, and ATF4, demonstrating that PERK is essential for melatonin's effect on the UPR response. How melatonin interacts with PERK remains unclear. A study showed that pretreatment with luzindole (a melatonin receptor antagonist targeting MT1 and MT2) did not prevent melatonin from enhancing thapsigargin‐induced CHOP expression in renal cancer cells, suggesting a potential receptor‐independent mechanism [68]. Similarly, Lang et al. (2021) proposed that melatonin activates the PERK‐ATF4 pathway independently of its receptors, as MT1 was absent in their head and neck cancer models [62]. However, other studies suggest that melatonin may act through receptor‐dependent mechanisms [62, 69, 70]. These findings highlight a complex interplay between melatonin and the PERK pathway, warranting further investigation.

We observed that melatonin induces early apoptosis in BeWo cells but has no effect on late apoptosis. The proapoptotic effects of melatonin have been evaluated in various cancer cell lines using flow cytometry with Annexin V‐PI staining [13, 18, 63, 71, 72, 73, 74, 75, 76, 77, 78, 79, 80, 81, 82, 83]. While some studies reported an increase in total apoptosis, they did not distinguish between early and late apoptotic stages [13, 18, 63, 71, 72, 73, 74, 75]. Other studies that have examined both early and late apoptosis report inconsistent findings potentially due to difference in cell types, melatonin concentration and exposure duration [76, 77, 78, 79, 80, 81, 82, 83]. In fact, in neuroblastoma cells, melatonin promotes early apoptosis and induces differentiation, reducing proliferation and the initial stages of carcinogenesis [84]. Annexin V binds to externalized phosphatidylserine, an early marker of apoptosis and a process essential for cell differentiation [85]. Previous work has shown that melatonin regulates Human chorionic gonadotropin beta chain (hCG‐β) secretion in BeWo cells, a key factor in trophoblast differentiation [20, 86, 87]. The initiation of early apoptosis and hCG‐β regulation by melatonin may suggest a role in modulating BeWo cell differentiation and affecting tumor progression. More research needs to be done on the subject.

Melatonin has been shown to trigger different apoptotic signaling pathways depending on the cancer model [47]. For example, Li et al. (2022) reported that melatonin's proapoptotic actions occur through NF‐κB and IκB modulation in gastric cancer cells, alongside involvement of the PERK pathway [63]. In contrast, our data suggest that in BeWo trophoblastic cells, melatonin's effects are more specifically associated with the PERK pathway, rather than with NF‐κB or IκB. This contrast underscores the need to better understand the mechanisms behind melatonin's proapoptotic effects, especially in specific cancer models pathway. Our previous work has shown that melatonin contributes to BeWo cell death by inducing intrinsic apoptosis and downregulating autophagy [35, 38]. Consistent with these findings, the present study shows that melatonin increases the expression of Bax, a key protein in intrinsic apoptosis, as well as cl‐PARP. The apoptotic effect of melatonin is mediated through the PERK‐CHOP pathway in various carcinoma cell types [33, 43]. CHOP induces apoptosis by modulating Bcl‐2 and Bax levels [88, 89]. In our study, the induction of early apoptosis by melatonin was lost when using PERK‐specific siRNA or scramble siRNA, suggesting that transfection may have induced a protective pathway in BeWo cells. For example, siRNA transfection can activate autophagy that may help sustain cell viability [90].

Both the presence of melatonin and the absence of PERK appear detrimental to BeWo choriocarcinoma cells. Knockdown of PERK increased early and total apoptosis in BeWo cells treated with tunicamycin compared to scramble siRNA‐transfected cells. This could be due to PERK deficiency leading to increased ROS accumulation and reduced NRF2 activation [91, 92, 93]. As tunicamycin induces oxidative stress, the absence of PERK may lead to reduced NRF2 activation, impairing the ability of BeWo cells to cope with the oxidative challenge. This could explain why PERK appears to contribute to BeWo cell survival, as observed in other cancer types [91, 92, 93]. PERK has been associated with poor prognosis in breast and thyroid cancers, potentially influencing tumor microenvironment and immune cell infiltration [94]. Suppression of PERK signaling has been shown to impede tumor growth and increase tumor sensibility to chemotherapy in colon cancer [95]. In colon and lung cancer, activation of p‐eIF2α and ATF4 signaling increases resistance to chemotherapy [96, 97]. More specifically, ATF4 overexpression leads to multidrug resistance, while ATF4 knockout reduces glutathione synthesis and increases chemotherapy sensitivity in lung cancer [97]. ER stress appears to be a pathway that enables cancer cells to resist apoptosis induced by chemotherapeutic agents. In support of this, PERK inhibitors have been shown to supress tumor development [61]. Melatonin may also help attenuate this resistance by inhibiting PERK pathway [98]. Fan et al. demonstrated that pretreatment with tunicamycin and melatonin increased doxorubicin‐induced apoptosis in hepatic carcinoma cells, whereas tunicamycin alone reduces doxorubicin‐induced apoptosis in liver cancer cells [98]. Other studies have shown that melatonin enhances cytotoxic effects of chemotherapeutic agents in cancer cells, increasing hepatocellular carcinoma sensitivity to sorafenib via the PERK‐ATF4‐Beclin1 pathway [33, 99].

## 5 Conclusion

This study demonstrates that melatonin activates the PERK arm of the UPR pathway and induces early apoptosis in BeWo cells. Moreover, PERK deficiency increases the susceptibility of these cells to apoptosis. Our findings highlight the role of melatonin and the PERK‐UPR pathway in promoting cancer cell apoptosis. Targeting UPR signaling to disrupt the balance between adaptive ER stress and ER stress‐induced apoptosis through melatonin could offer a novel therapeutic approach for treating cancers, including choriocarcinoma. Future studies should explore the potential of melatonin as an adjuvant therapy, potentially amplifying ER stress and apoptosis to enhance therapeutic efficacy in cancer treatment while protecting healthy cells.

## Author Contributions

All co‐authors contributed to the content and discussion of each section of the manuscript. Josianne Bienvenue‐Pariseault and Cathy Vaillancourt contributed to the design of the study protocol. Josianne Bienvenue‐Pariseault, Philippe Wong‐Yen, and Darius Stakamatos were involved in data acquisition. Lucas Sagrillo‐Fagundes, Marie Cohen and Cathy Vaillancourt were actively involved in the revising of the manuscript. Josianne Bienvenue‐Pariseault wrote the original version and created the figures of the manuscript. All co‐authors provided critical revisions and their final approval for the manuscript's publication and all authors agree with the contents of this manuscript.

## Conflicts of Interest

The authors declare no conflicts of interest.

## Supporting information

Supporting data.

## Data Availability

Data available on request from the authors.
