# Supplementary material for: Melatonin Induces PERK‐ATF4 Unfolded Protein Response and Apoptosis in Human Choriocarcinoma Cells
Source: J Pineal Res. 2025 Aug 27;77(5):e70072. doi: 10.1111/jpi.70072 (PMC12391747; doi:10.1111/jpi.70072)
Supplement: Supplementary file 1 — Supporting data. [file JPI-77-e70072-s001.pdf]

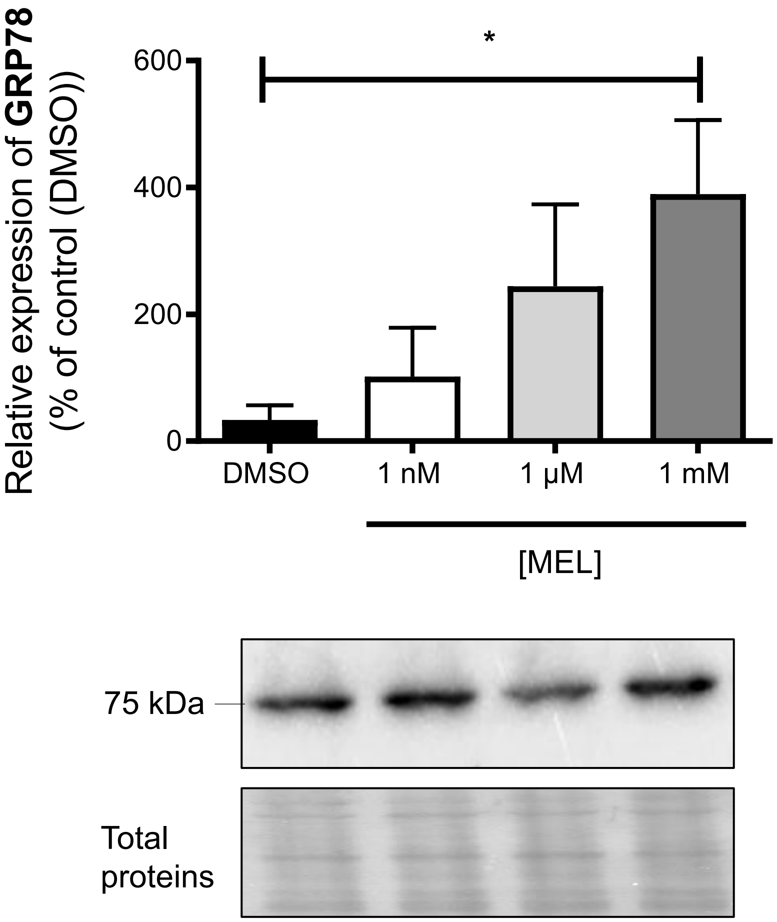

Supplementary data 1. Melatonin at 1 mM increases protein expression of GRP78. BeWo cells were treated with the vehicle (DMSO) or melatonin (1 mM, 1 uM or 1 mM) or Tm (3,5 ug/mL) under 8% of oxygen during 24-h. GRP78 protein expression was determine by Western blot. Equal protein amounts of cell lysates were subjected to Western blot assay using anti-GRP78. Total protein was using for normalization. GRP78: 78-kDa glucose-regulated protein; DMSO: dimethylsulfoxyde; Mel: melatonin; Tm: tunicamycin. Data are shown as mean ± SD and were analyzed using ANOVA (*P < 0.05), n=4.

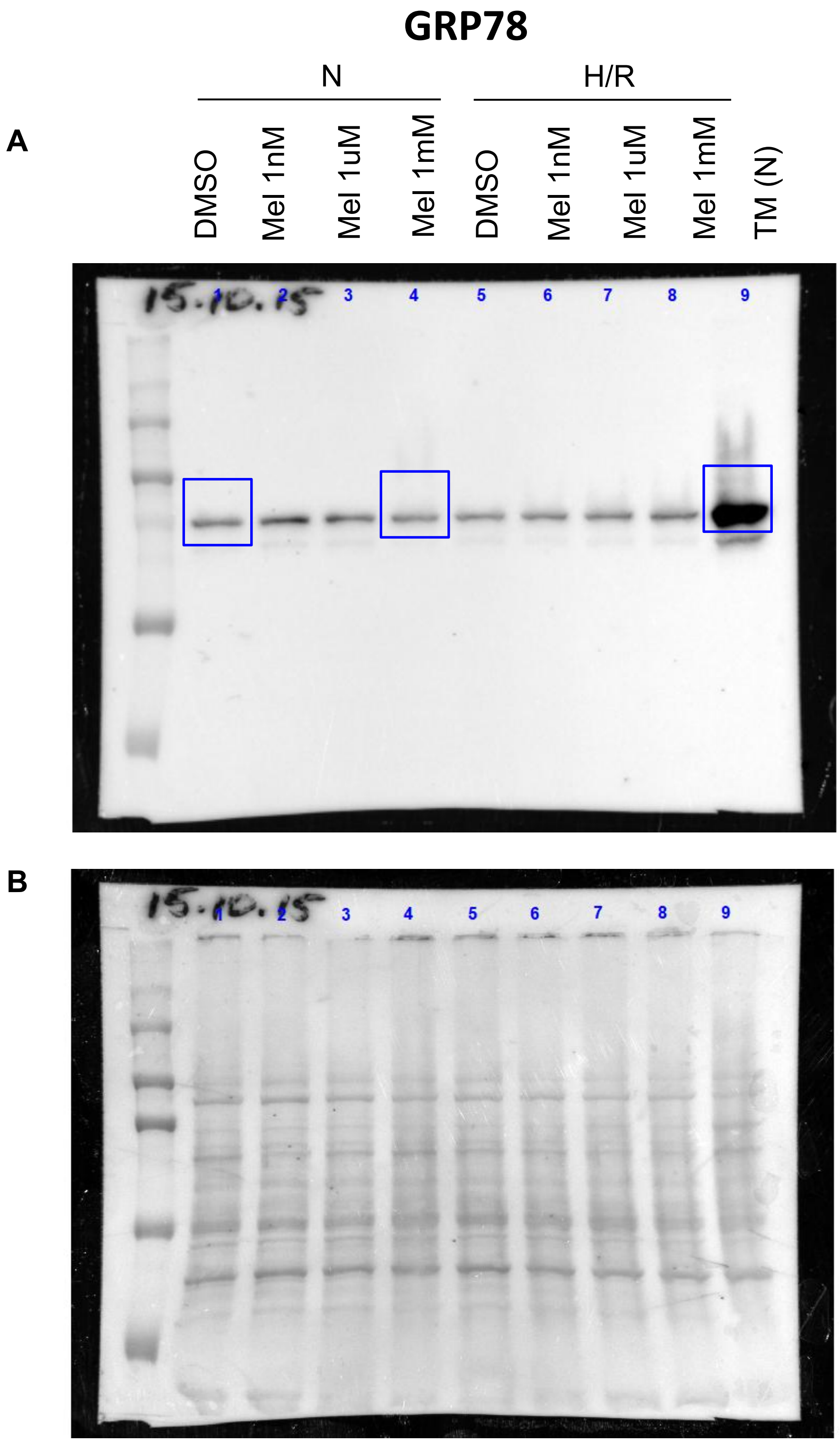

Supplementary data 2. This is the original blot of Fig. 1**.** Melatonin increases endoplasmic reticulum stress in BeWo cells. BeWo cells were treated with the vehicle (DMSO) or melatonin (1 mM, 1 uM, 1 nM) or TM (3,5 ug/mL) under 8% of oxygen during 24-h (normoxia) or under 0,5% of oxygen for 4-h followed by an incubation under 8% of oxygen during 22-h (hypoxia/reoxygenation). A: GRP78 protein expression was determine by Western blot. Equal protein amounts of cell lysates were subjected to Western blot assay using anti-GRP78. Blue boxes mark the bands shown in Fig. 1. B: Total protein was using for normalization. GRP78: 78-kDa glucose-regulated protein; N: Normoxia; H/R: Hypoxia/Reoxygenation; DMSO: dimethylsulfoxyde; Mel: Melatonin; Tm: Tunicamycin.

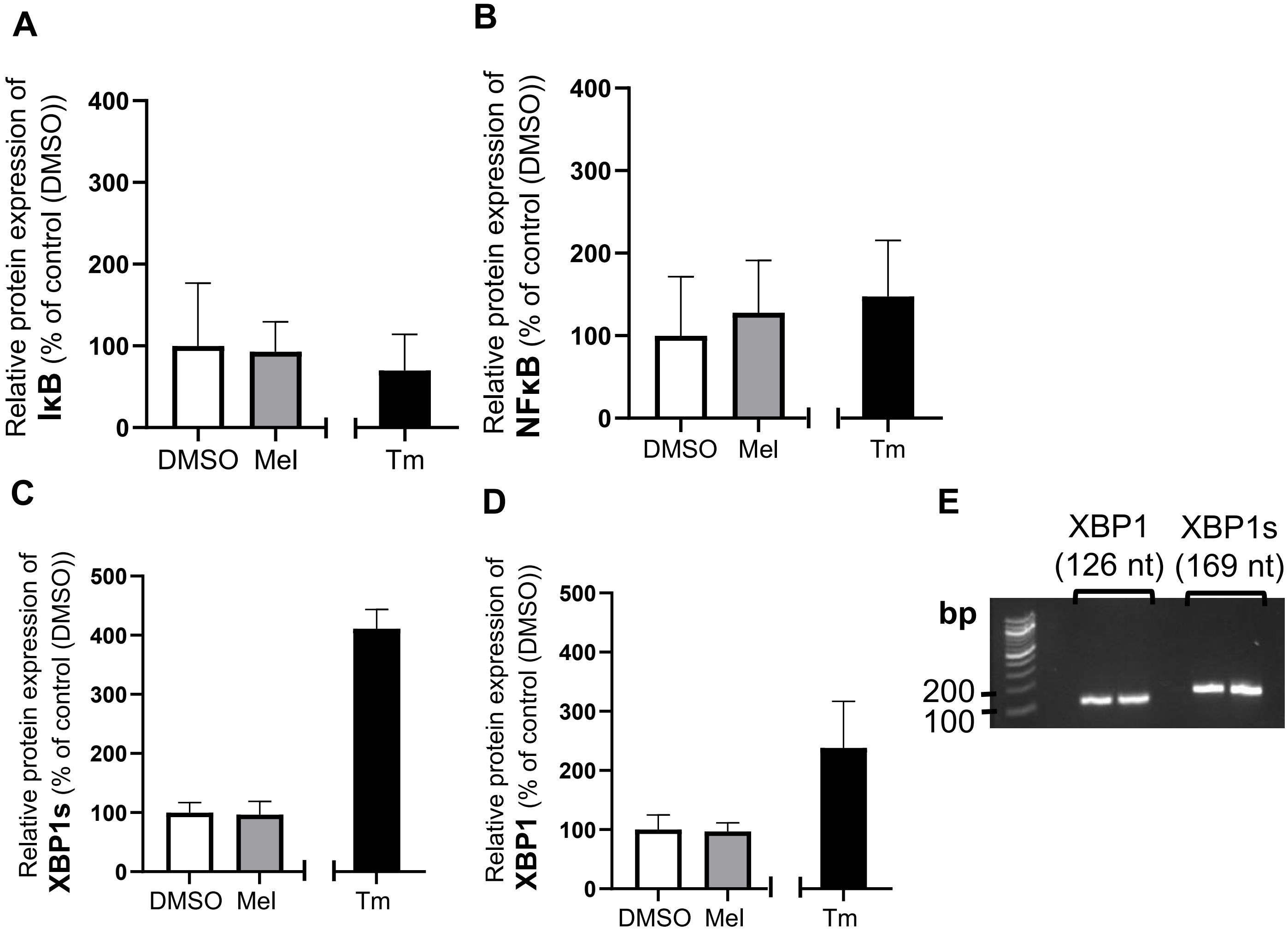

Supplementaty data 3. Melatonin has no effect on the protein level of factors implicated in IREα-UPR pathway. BeWo cells were treated with the vehicle (DMSO 0.1%) or melatonin (1 mM) or Tm (3.5 μg/mL) under normoxia (8% O_2) conditions during 24-h. A, B: IkB and NFkB ratio protein level was determined by Western blot. Equal protein amounts of cell lysates (35 μg) were subjected to Western blot assay using anti-NFkB and anti-IkB. Total protein was used for normalization. NF-κB and IκB relative protein expression data displayed separately in this supplementary figure were used to generate the NF-κB/IκB ratio presented in Figure 2C of the main manuscript. C, D: XBP1s and XBP1 gene expression was determined by RT-qPCR using specific primers. *B2M* and *SDHA* were used as references genes for normalization. XBP1 and XBP1s relative gene expression data displayed separately in this supplementary figure were used to generate the XBP1s/XBP1 ratio presented in Figure 2D of the main manuscript. E: XBP1 and XBP1s amplicons obtained by RT-qPCR from a pool of treatment had expected size. DMSO: dimethylsulfoxyde; IkB: inhibitor of nuclear factor kappa B; IREα: serine/threonine-protein kinase/endoribonuclease inositol-requiring enzyme 1α; Mel: melatonin; NFkB: nuclear factor-kappa B; Tm: tunicamycin; XBP1: X-box binding protein 1; XBP1s: spliced X-box binding protein 1; Data are shown as mean ± SD and were analyzed using an unpaired t-test (DMSO vs Mel, $^*p < 0.05$), n=4-5.

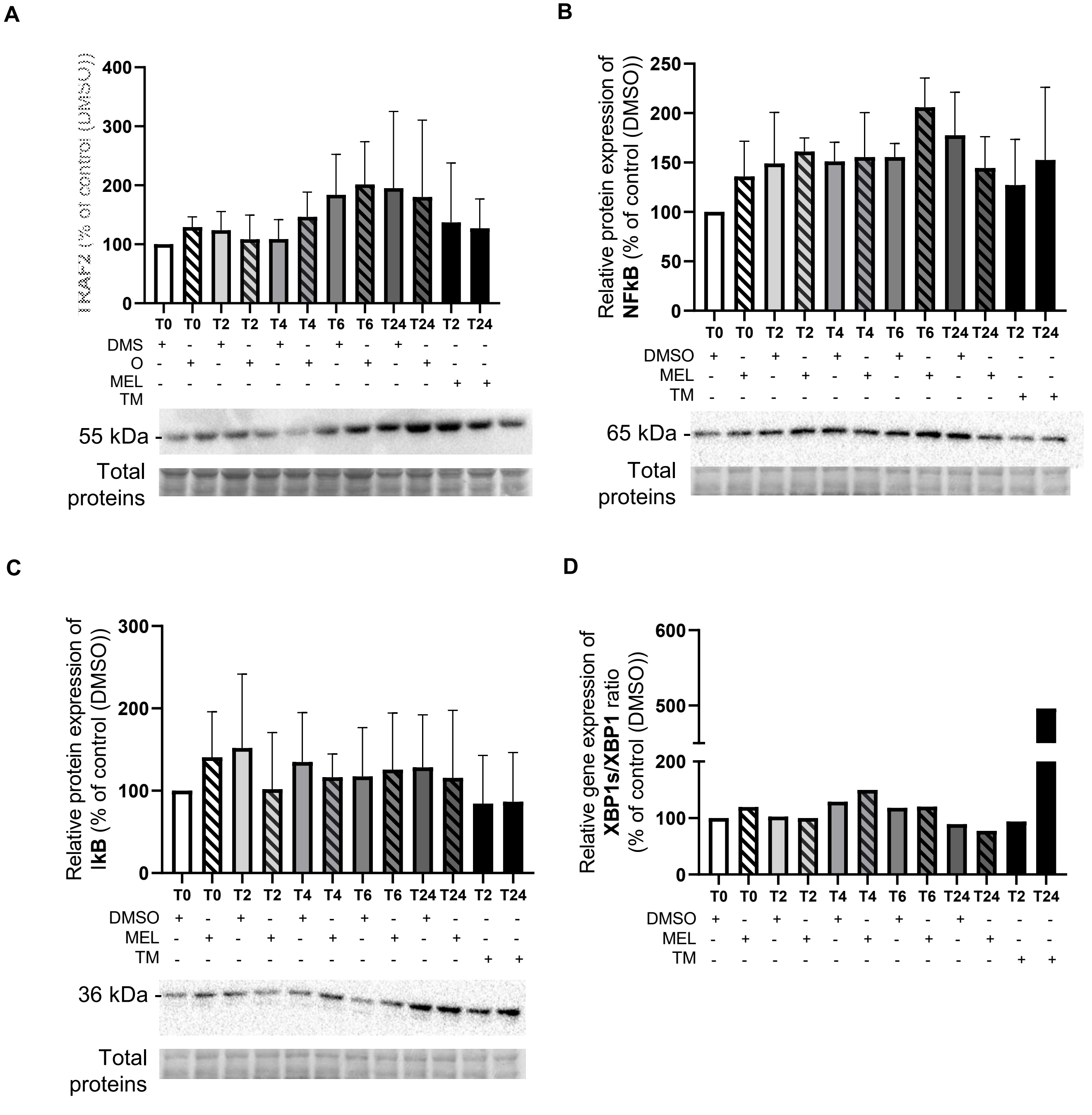

Supplementary data 4. Effect of time course melatonin treatment on IRE1a-UPR pathway's factors protein expression. BeWo cells were treated with the vehicle (DMSO 0,1%) or melatonin (1 mM) or Tm (3,5 ug/mL) under normoxia (8% O_2) conditions during 0-h, 2-h, 4-h, 6-h or 24-h. A, B, C: A: TRAF2, NFkB and IkB protein expression was determined by Western blot. Equal protein amounts of cell lysates were subjected to Western blot assay using anti-TRAF2, anti-NFkB and anti-IkB. B: Total protein was used for normalization. D: *XBP1s/XBP1* gene expression was determine by RT-qPCR using specific primers. B2M and SDHA sequences were used for normalization. TRAF2: TNF receptor-associated factor 2; NFkB: nuclear factor-kappa B; IkB: inhibitor of nuclear factor kappa B; XBP1: X-box binding protein 1; XBP1s: Spliced X-box binding protein 1; DMSO: dimethylsulfoxyde; Mel: melatonin; Tm: tunicamycin. Data are shown as mean ± SD and were analyzed using an unpaired t-test (DMSO vs Mel, *P < 0.05), (A-C) n = 4, (D) n = 1.

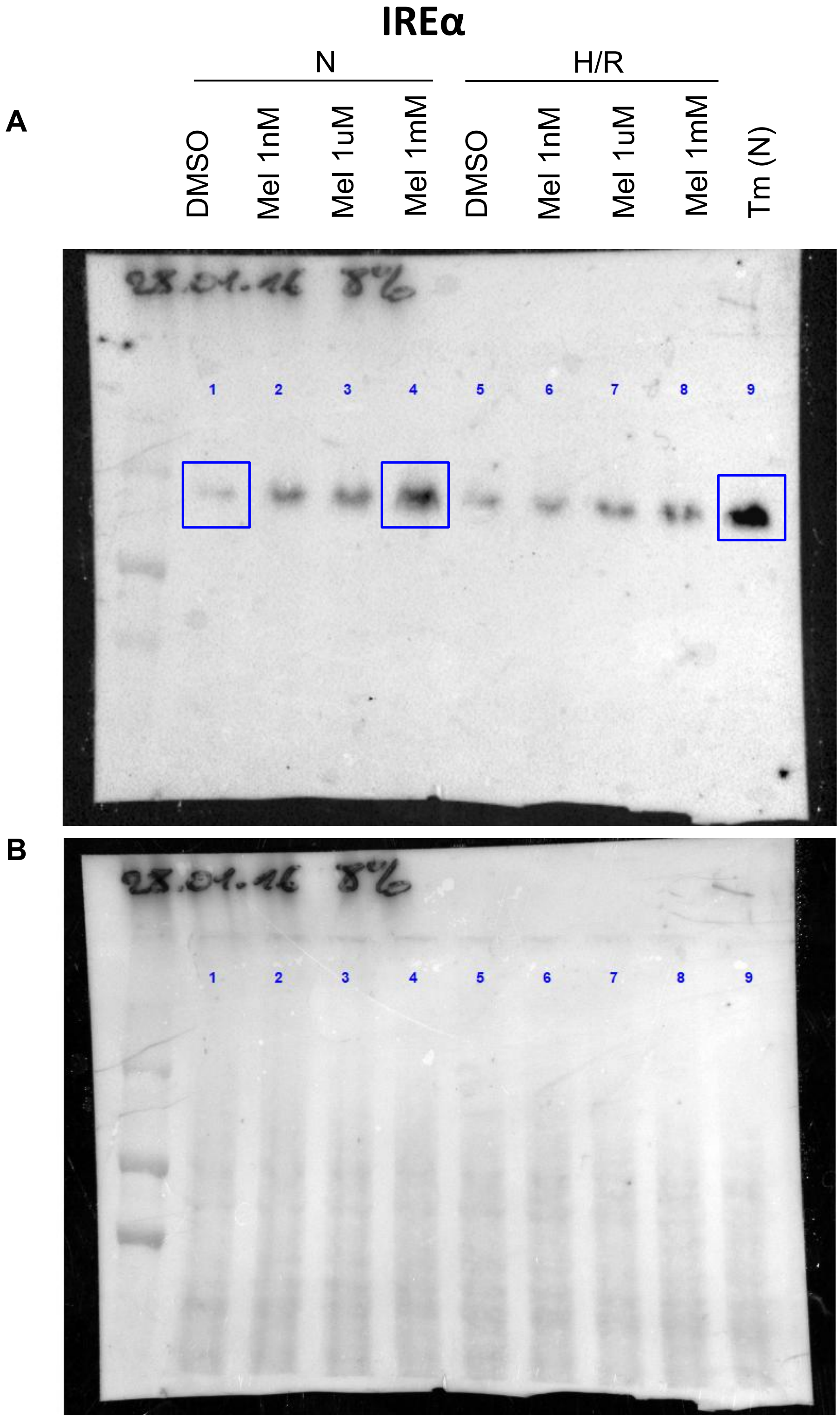

Supplementary data 5. This is the **o**riginal blot of Fig. 2A**.** Melatonin doesn't affect the protein level of the downstream factors of IREα-UPR pathway. BeWo cells were treated with the vehicle (DMSO) or melatonin (1 mM, 1 uM or 1 nM) or TM (3,5 ug/mL) under 8% of oxygen during 24-h (normoxia) or under 0,5% of oxygen for 4-h followed by an incubation under 8% of oxygen during 22-h (hypoxia/reoxygenation). A: IREα protein expression was determined by Western blot. Equal protein amounts of cell lysates were subjected to Western blot assay using anti-IREα. Blue boxes mark the bands shown in Fig. 2A. B: Total protein was using for normalization. IREα: serine/threonine-protein kinase/endoribonuclease inositol-requiring enzyme 1 α; N: Normoxia; H/R: Hypoxia/Reoxygenation; DMSO: dimethylsulfoxyde; Mel: Melatonin; Tm: Tunicamycin.

TRAF2

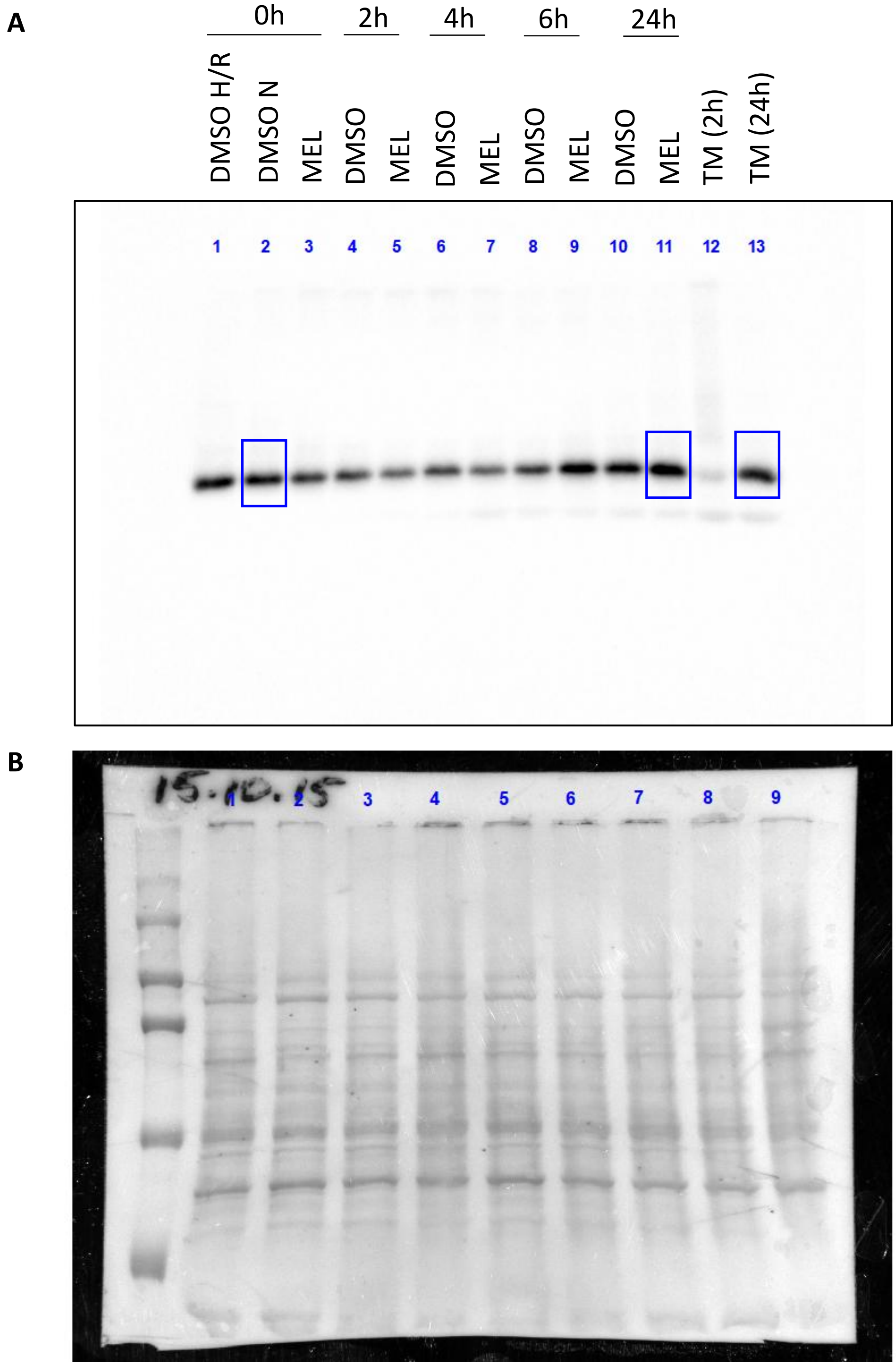

Supplementary data 6. This is the original blot of Fig. 2B. Melatonin doesn't affect the protein level of the downstream factors of IREα-UPR pathway. BeWo cells were treated with the vehicle (DMSO) or melatonin (1 mM) or TM (3,5 ug/mL) under 8% of oxygen during 0-h, 2-h, 4-h, 6-h or 24-h. A: TRAF2 protein expression was determined by Western blot. Equal protein amounts of cell lysates were subjected to Western blot assay using anti-TRAF2. Blue boxes mark the bands shown in Fig. 2B. B: Total protein was using for normalization. TRAF2: TNF receptor-associated factor 2; N: Normoxia; H/R: Hypoxia/Reoxygenation; DMSO: dimethylsulfoxyde; MEL: Melatonin; TM: Tunicamycin.

NFkB

A

B

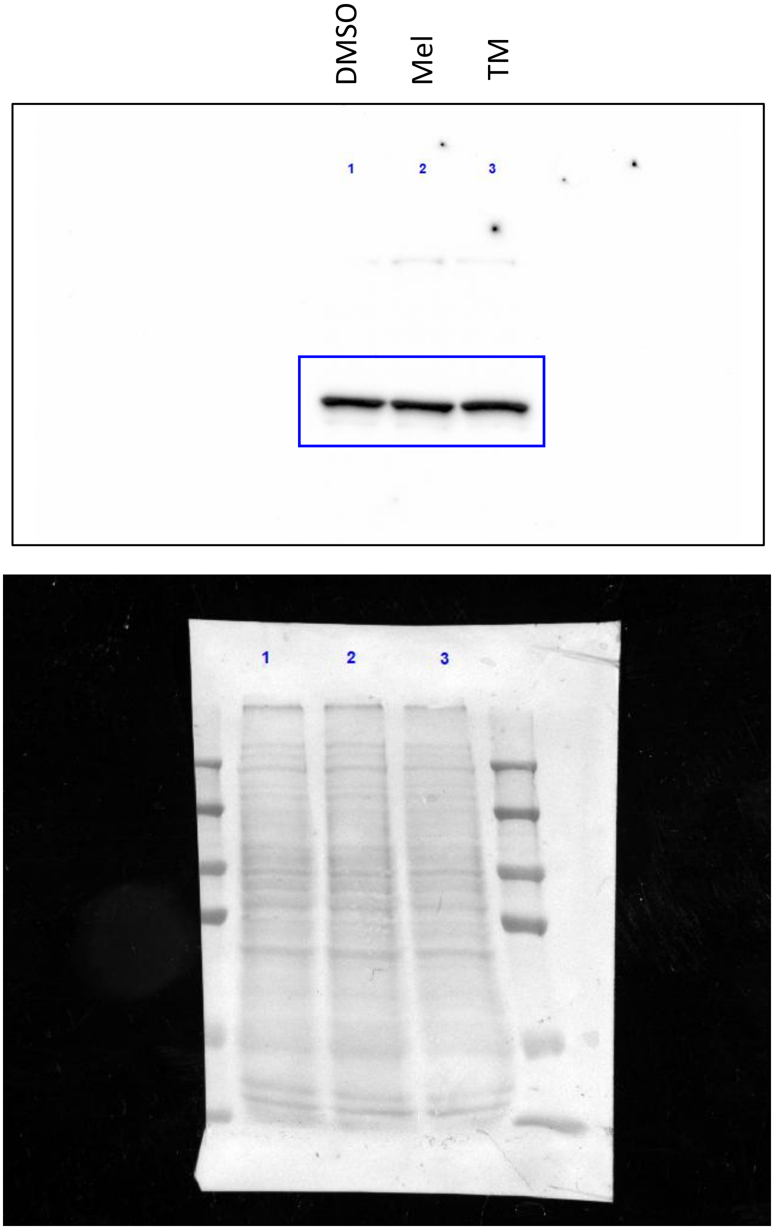

Supplementary data 7. This is the original blot of Fig. 2C. Melatonin doesn't affect the protein level of the downstream factors of IREα-UPR pathway. BeWo cells were treated with the vehicle (DMSO) or melatonin (1 mM) or TM (3,5 ug/mL) under 8% of oxygen during 24-h. A: NFkB protein expression was determined by Western blot. Equal protein amounts of cell lysates were subjected to Western blot assay using anti-NFkB. Blue boxes mark the bands shown in Fig. 2C. B: Total protein was using for normalization. NFkB: nuclear factor-kappa; N: Normoxia; H/R: Hypoxia/Reoxygenation; DMSO: dimethylsulfoxyde; MEL: Melatonin; TM: Tunicamycin.

IkB

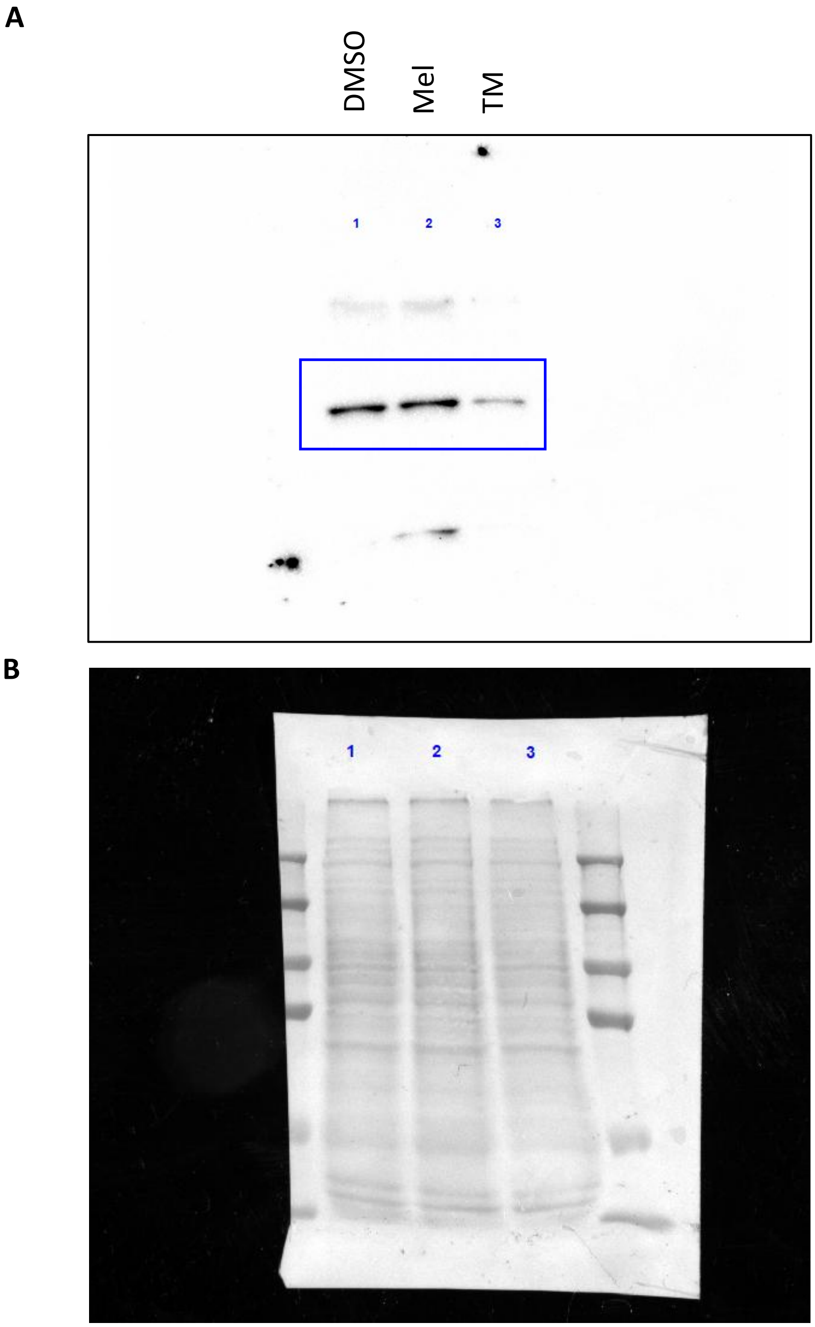

Supplementary data 8. This is the original blot of Fig. 2C. Melatonin doesn't affect the protein level of the downstream factors of IREα-UPR pathway. BeWo cells were treated with the vehicle (DMSO) or melatonin (1mM) or TM (3,5 ug/mL) under 8% of oxygen during 24h. A: IkB protein expression was determined by Western blot. Equal protein amounts of cell lysates were subjected to Western blot assay using anti-IkB. Blue boxes mark the bands shown in Fig. 2C. B: Total protein was using for normalization. ; IkB: inhibitor of nuclear factor kappa B; N: Normoxia; H/R: Hypoxia/Reoxygenation; DMSO: dimethylsulfoxyde; MEL: Melatonin; TM: Tunicamycin.

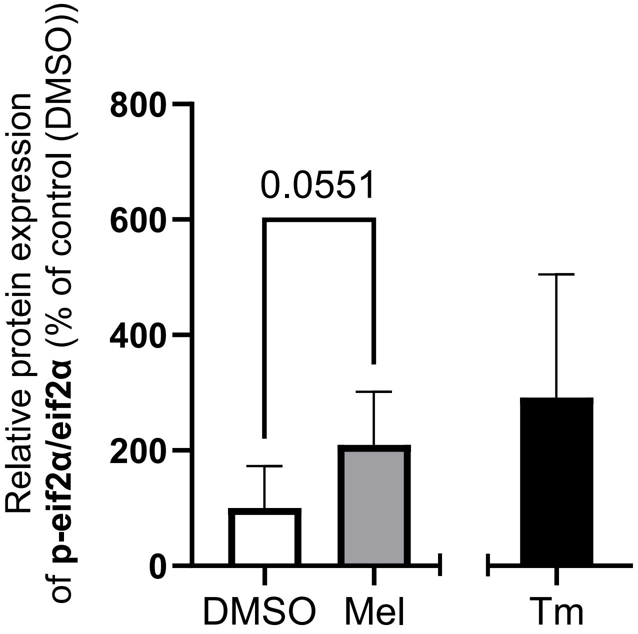

Supplementary data 9. Melatonin effect on p-eif2α/eif2α ratio. BeWo cells were treated with the vehicle (DMSO 0.1%) or melatonin (1 mM) or Tm (3.5 µg/mL) under normoxia (8% O_2) conditions during 24-h. P-eif2α and eif2α ratio protein level was determined by Western blot. Equal protein amounts of cell lysates (35 µg) were subjected to Western blot assay using anti-p-eif2α and anti-eif2α. Total protein was used for normalization. P-eif2α and eif2α relative protein expression data displayed separately in this supplementary figure were used to generate the p-eif2α/eif2α ratio presented in Figure 4A and B of the main manuscript. DMSO: dimethylsulfoxyde; eif2α: eukaryotic initiation factor 2; Mel: melatonin; P-eif2α: phosphorylation of the eukaryotic initiation factor 2; Tm: tunicamycin. Data are shown as mean ± SD and were analyzed using an unpaired t-test (DMSO vs. Mel, *$p < 0.05$), n=4-6.

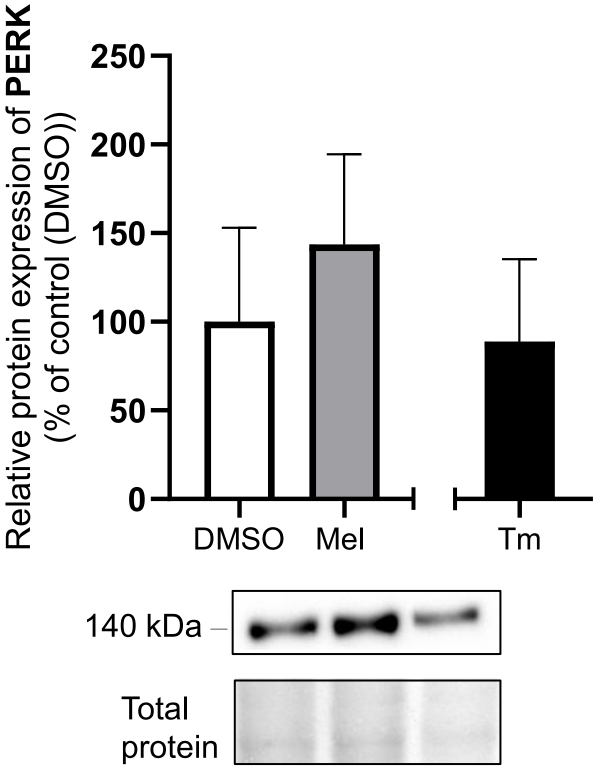

Supplementary data 10. Melatonin has no effect on PERK protein level. BeWo cells were treated with the vehicle (DMSO 0,1%) or melatonin (1mM) or TM (3,5 ug/mL) under normoxia (8% O_2) conditions during 24-h. PERK protein level was determined by Western blot. Equal protein amounts of cell lysates were subjected to Western blot assay using anti-PERK. Total protein was using for normalization. DMSO: dimethylsulfoxyde; Mel: melatonin; Tm: tunicamycin. Data are shown as mean ± SD and were analyzed using an unpaired t-test (DMSO vs Mel, *P < 0.05), n=5.

A

Primers	Primer sequences (5' -> 3')	Amplicon size (nt)	References
**CHOP**	Foward : AAGGCACTGAGCGTATCATGT<br>Reverse : TGAAGATACACTTCCTTCTTGAACA	105	(Luke R.G. et al 2013)
**ATF4**	Foward :CCCTAGTCCAGGAGACTAATAAGCA<br>Reverse : ACTTTCTGGGAGATGGCCAAT	68	(Baoquin et al., 2009)
**B2M**	Foward : GATGAGTATGCCTGCCGTGT<br>Reverse : CTGCTTACATGTCTCGATCCCA	79	Primer Blast
**SDHA**	Foward : TACAAGGTGCGGATTGATG<br>Reverse : CGATCACGGGTCTATATTCAA	148	Primer Blast

B

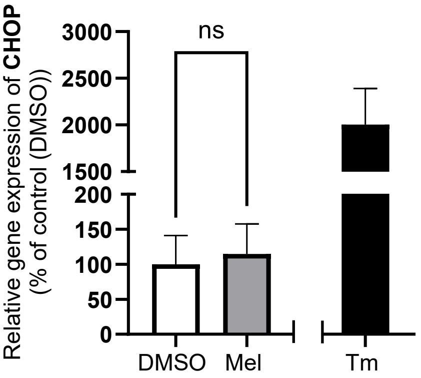

C

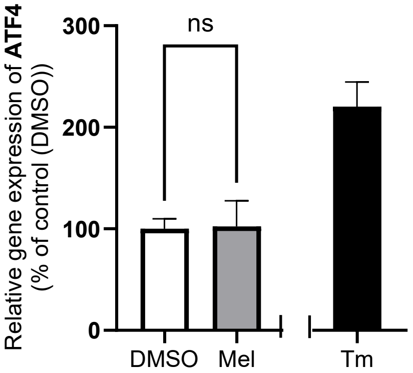

D

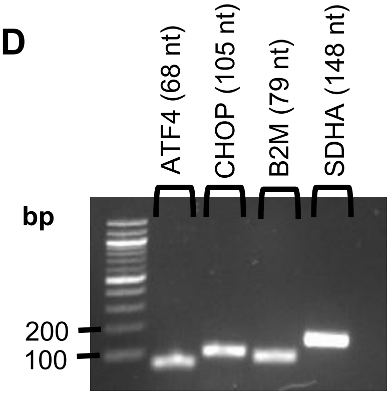

Supplementary data 11. Melatonin has no effect on CHOP and ATF4 gene expression. BeWo cells were treated with the vehicle (DMSO 0,1%) or melatonin (1 mM) or TM (3,5 ug/mL) under normoxia (8% O_2) conditions during 24-h. A: Primers used for RT-qPCR. B, C: CHOP and ATF4 gene expression was determine by RT-qPCR using specific primers. B2M and SDHA sequences were used for normalization. D: ATF4, CHOP, B2M and SDHA amplicon obtained by RT-qCR. ATF4: Activating Transcription Factor 4; CHOP: DNA damage-inducible transcript 3, also known as C/EBP homologous protein; Bax: Bcl-2–associated X; Bcl-2: B-cell lymphoma 2; B2M : Beta-2-microglobubin; SDHA : Succinate Dehydrogenase Complex Flavoprotein Subunit A; DMSO: dimethylsulfoxyde; Mel: melatonin; Tm: tunicamycin. Data are shown as mean ± SD and were analyzed using an t-test (DMSO vs Mel, *P < 0.05), n=4.

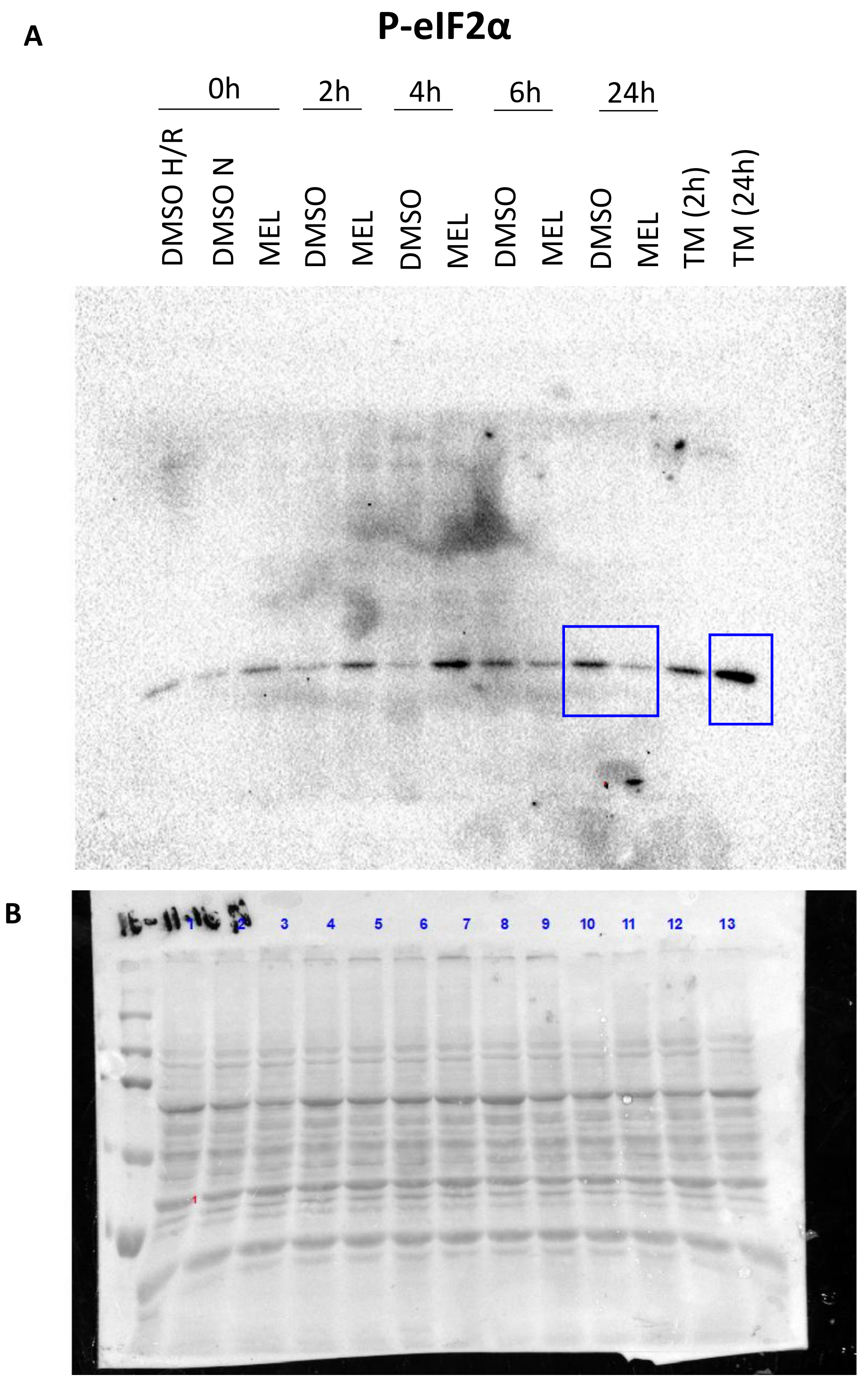

Supplementary data 12. This is the original blot of Fig. 4A. Melatonin increases the protein level of the downstream factors of PERK-UPR pathway. BeWo cells were treated with the vehicle (DMSO 0,1%) or melatonin (1mM) or TM (3,5 ug/mL) under 8% of oxygen during during 0h, 2h, 4h, 6h or 24h. A: p-eIF2α protein expression was determined by Western blot. Equal protein amounts of cell lysates were subjected to Western blot assay using anti-p-eIF2α. Blue boxes mark the bands shown in Fig. 4A. B: Total protein was used for normalization. P-eIF2α: Phosphorylation of the eukaryotic initiation factor 2 subunit alpha; DMSO: dimethylsulfoxyde; MEL: Melatonin; TM: Tunicamycin.

eIF2α

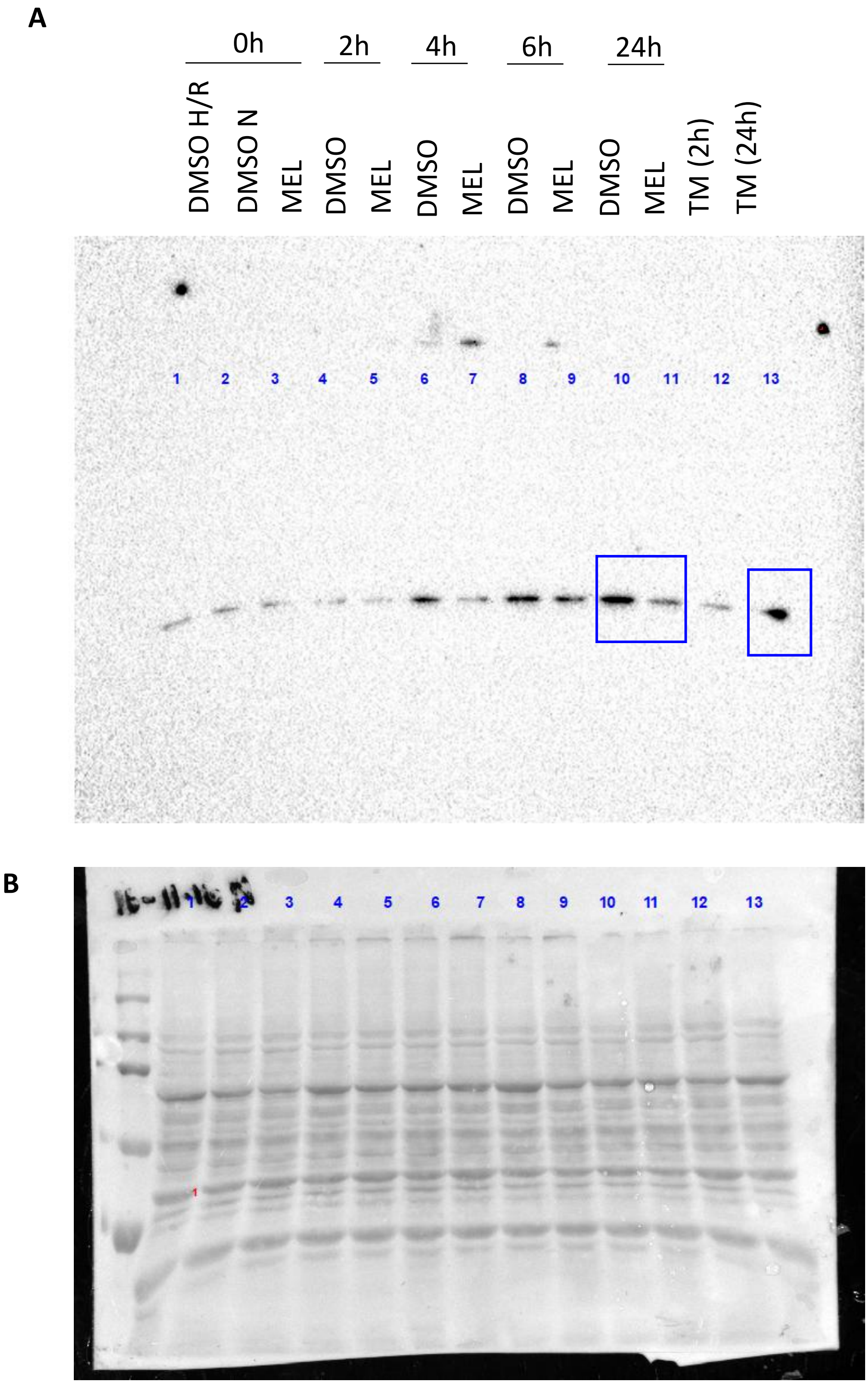

Supplementary data 13. This is the original blot of Fig. 4B. Melatonin increases the protein level of the downstream factors of PERK-UPR pathway. BeWo cells were treated with the vehicle (DMSO 0,1%) or melatonin (1mM) or TM (3,5 ug/mL) under 8% of oxygen during during 0h, 2h, 4h, 6h or 24h. A: eIF2α protein expression was determined by Western blot. Equal protein amounts of cell lysates were subjected to Western blot assay using anti-eIF2α. Blue boxes mark the bands shown in Fig. 4B. B: Total protein was used for normalization. eIF2α: Eukaryotic initiation factor 2 subunit alpha; DMSO: dimethylsulfoxyde; MEL: Melatonin; TM: Tunicamycin.

ATF4

A

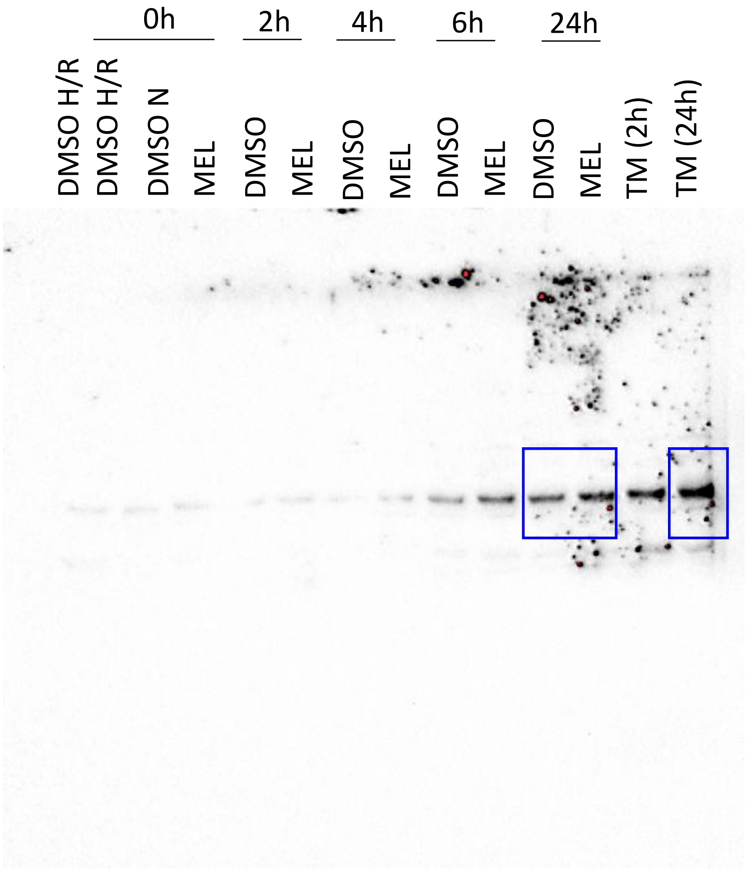

B

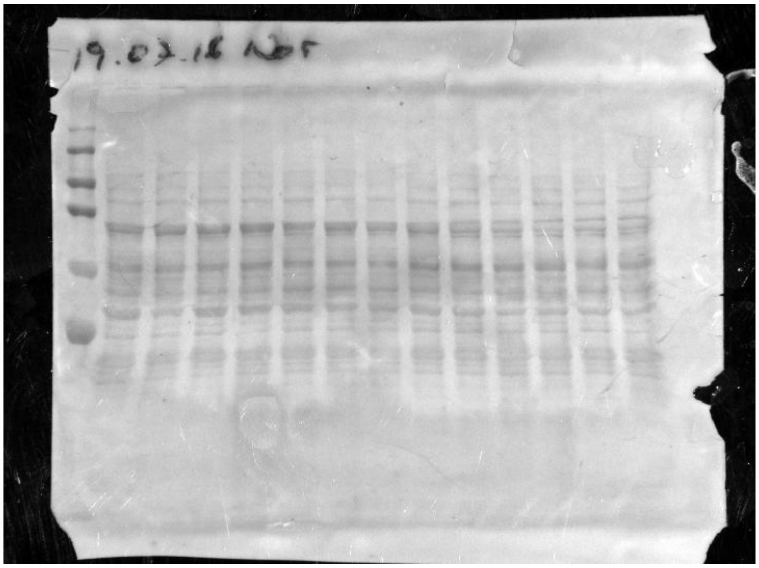

Supplementary data 14. This is the original blot of Fig. 4C. Melatonin increases the protein level of the downstream factors of PERK-UPR pathway. BeWo cells were treated with the vehicle (DMSO 0,1%) or melatonin (1mM) or TM (3,5 ug/mL) under 8% of oxygen during during 0h, 2h, 4h, 6h or 24h. A: ATF4 protein expression was determined by Western blot. Equal protein amounts of cell lysates were subjected to Western blot assay using anti-ATF4. Blue boxes mark the bands shown in Fig. 4C. B: Total protein was used for normalization. ATF4: Activating Transcription Factor 4; DMSO: dimethylsulfoxyde; MEL: Melatonin; TM: Tunicamycin.

CHOP

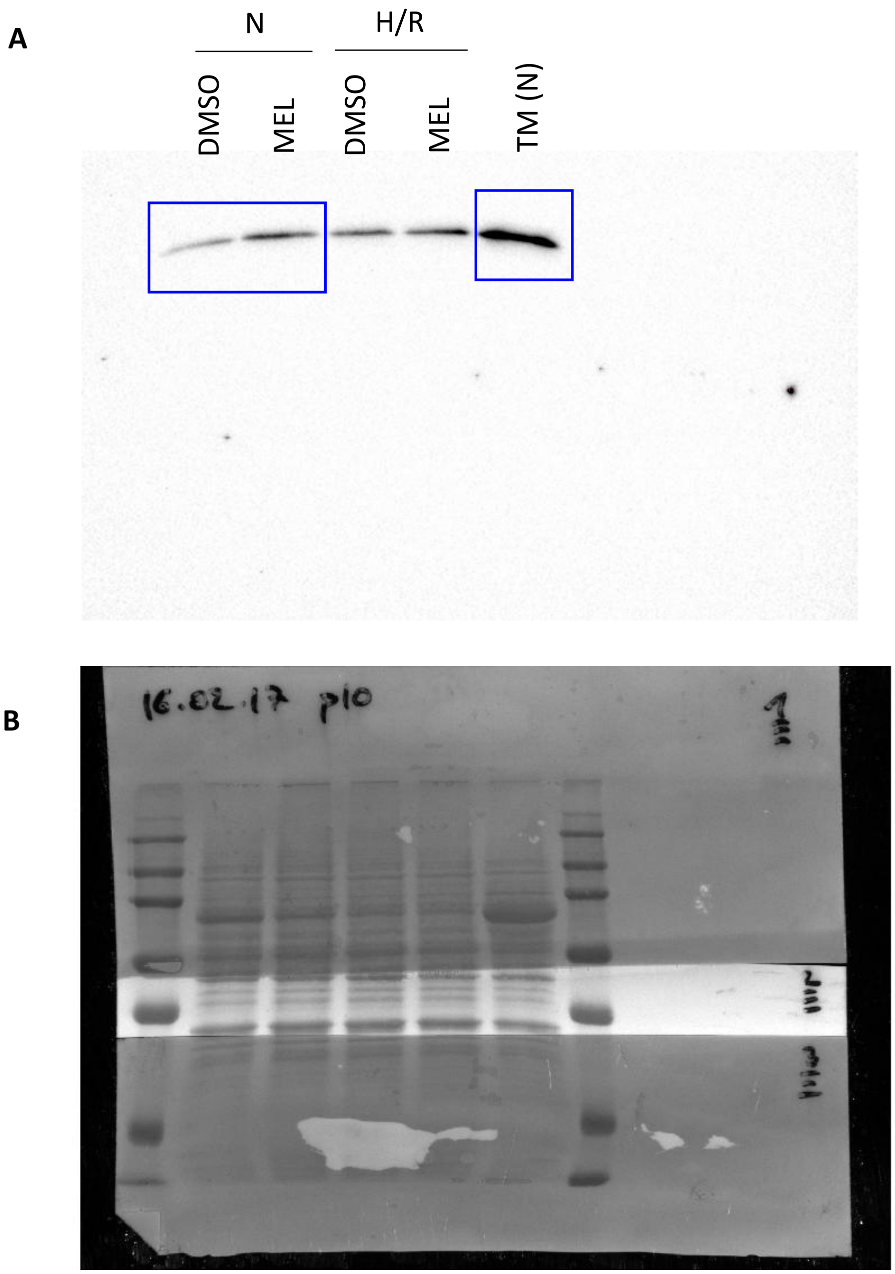

Supplementary data 15. This is the original blot of Fig. 4D. Melatonin increases the protein level of the downstream factors of PERK-UPR pathway. BeWo cells were treated with the vehicle (DMSO 0,1%) or melatonin (1mM) or TM (3,5 ug/mL) under under 8% of oxygen during 24h (normoxia) or under 0,5% of oxygen for 4h followed by an incubation under 8% of oxygen during 22h (hypoxia/reoxygenation). A: CHOP protein expression was determined by Western blot. Equal protein amounts of cell lysates were subjected to Western blot assay using anti-CHOP. Blue boxes mark the bands shown in Fig. 4D. B: Total protein was used for normalization. CHOP: DNA damage-inducible transcript 3; N: Normoxia; H/R: Hypoxia/Reoxygenation; DMSO: dimethylsulfoxyde; MEL: Melatonin; TM: Tunicamycin.

Bax

A

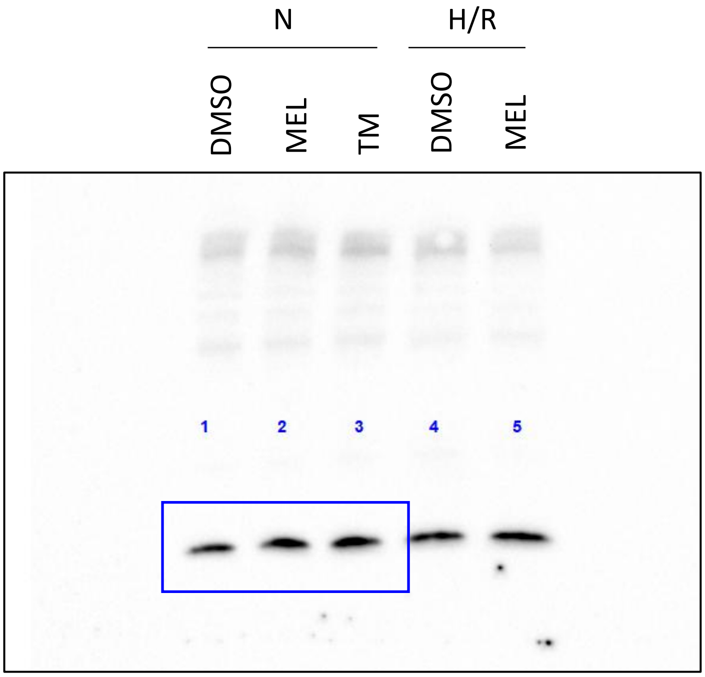

B

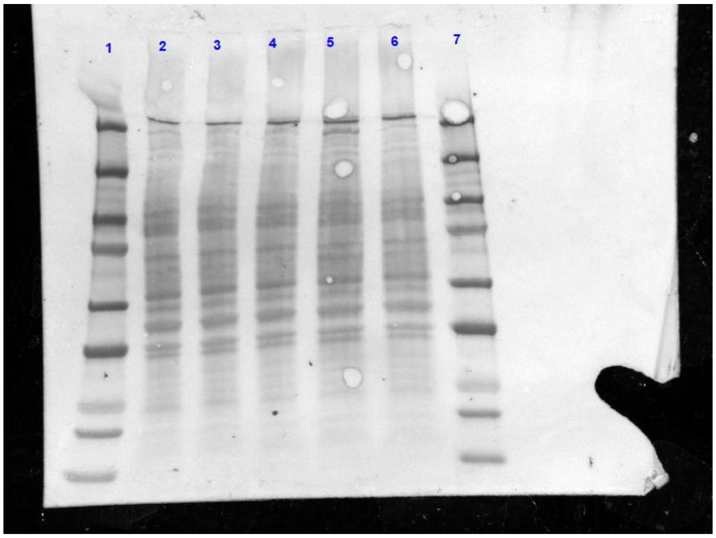

Supplementary data 16. This is the original blot of Fig. 4E. Melatonin increases the protein level of the downstream factors of PERK-UPR pathway. BeWo cells were treated with the vehicle (DMSO 0,1%) or melatonin (1mM) or TM (3,5 ug/mL) under under 8% of oxygen during 24h (normoxia) or under 0,5% of oxygen for 4h followed by an incubation under 8% of oxygen during 22h (hypoxia/reoxygenation). A: Bax protein expression was determined by Western blot. Equal protein amounts of cell lysates were subjected to Western blot assay using anti-Bax. Blue boxes mark the bands shown in Fig. 4E. B: Total protein was used for normalization. Bax: Bcl-2–associated X; N: Normoxia; H/R: Hypoxia/Reoxygenation; DMSO: dimethylsulfoxyde; MEL: Melatonin; TM: Tunicamycin

Bcl-2

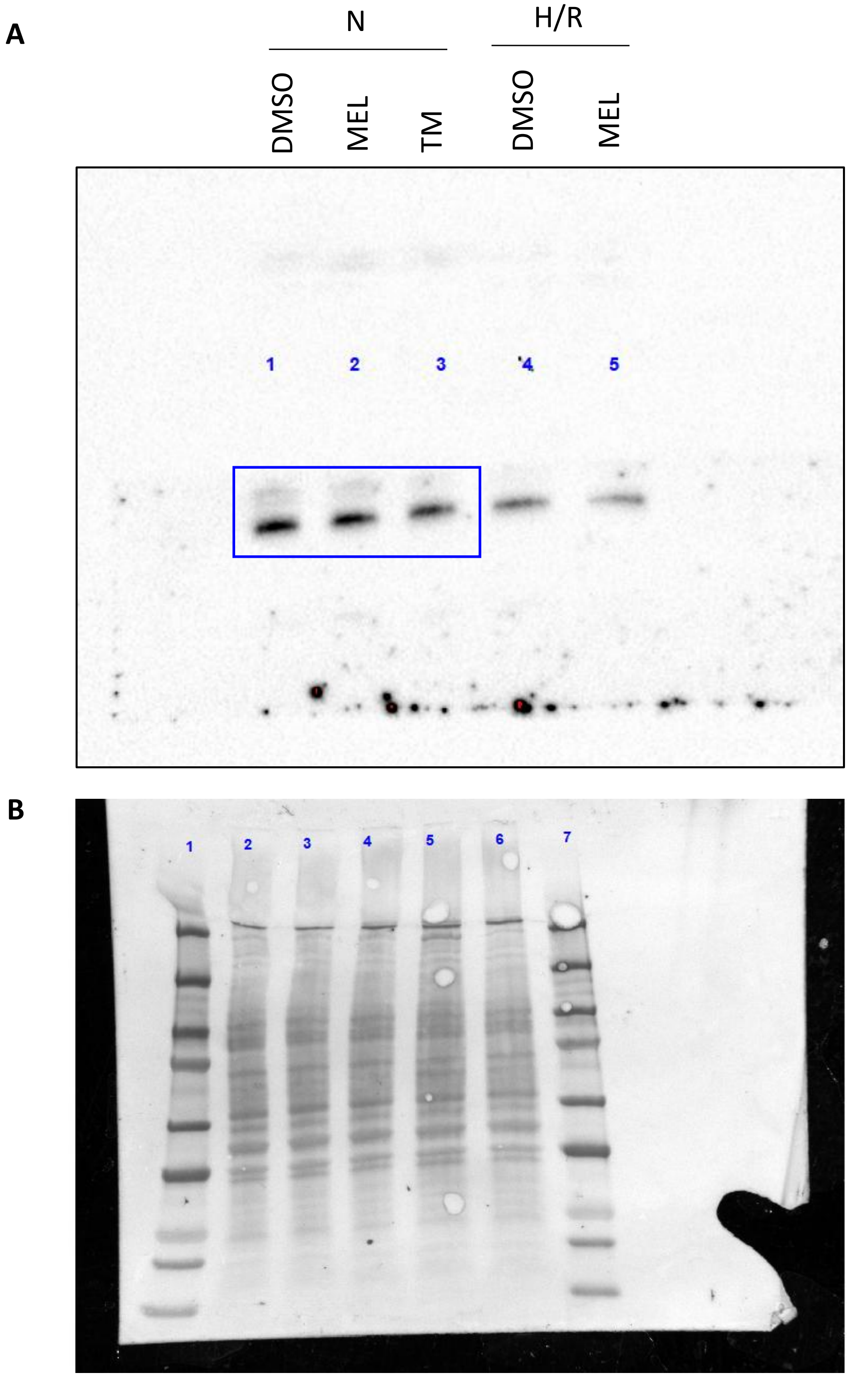

Supplementary data 17. This is the original blot of Fig. 4F. Melatonin increases the protein level of the downstream factors of PERK-UPR pathway. BeWo cells were treated with the vehicle (DMSO 0,1%) or melatonin (1mM) or TM (3,5 ug/mL) under under 8% of oxygen during 24h (normoxia) or under 0,5% of oxygen for 4h followed by an incubation under 8% of oxygen during 22h (hypoxia/reoxygenation). A: Bcl-2 protein expression was determined by Western blot. Equal protein amounts of cell lysates were subjected to Western blot assay using anti-Bcl-2. Blue boxes mark the bands shown in Fig. 4F. B: Total protein was used for normalization. Bcl-2: B-cell lymphoma 2; N: Normoxia; H/R: Hypoxia/Reoxygenation; DMSO: dimethylsulfoxyde; MEL: Melatonin; TM: Tunicamycin

Cl-PARP

A

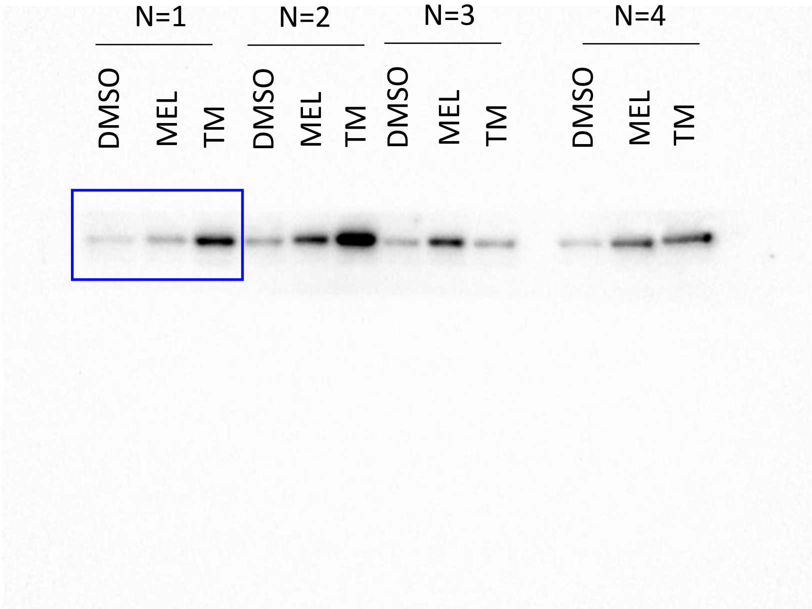

B

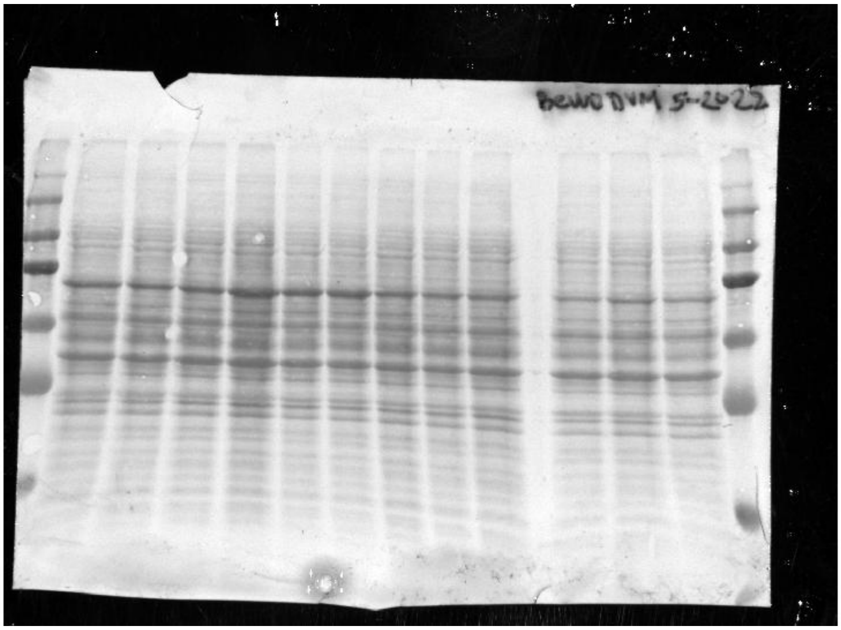

Supplementary data 18. This is the original blot of Fig. 4G. Melatonin increases the protein level of the downstream factors of PERK-UPR pathway. BeWo cells were treated with the vehicle (DMSO 0,1%) or melatonin (1mM) or TM (3,5 ug/mL) under under 8% of oxygen during 24h (normoxia). A: cl-Parp protein expression was determined by Western blot. Equal protein amounts of cell lysates were subjected to Western blot assay using anti-cl-Parp. Blue boxes mark the bands shown in Fig. 4G. Total protein was used for normalization. Cl-Parp: Cleaved poly(ADP-ribose) polymerase; DMSO: dimethylsulfoxyde; MEL: Melatonin; TM: Tunicamycin

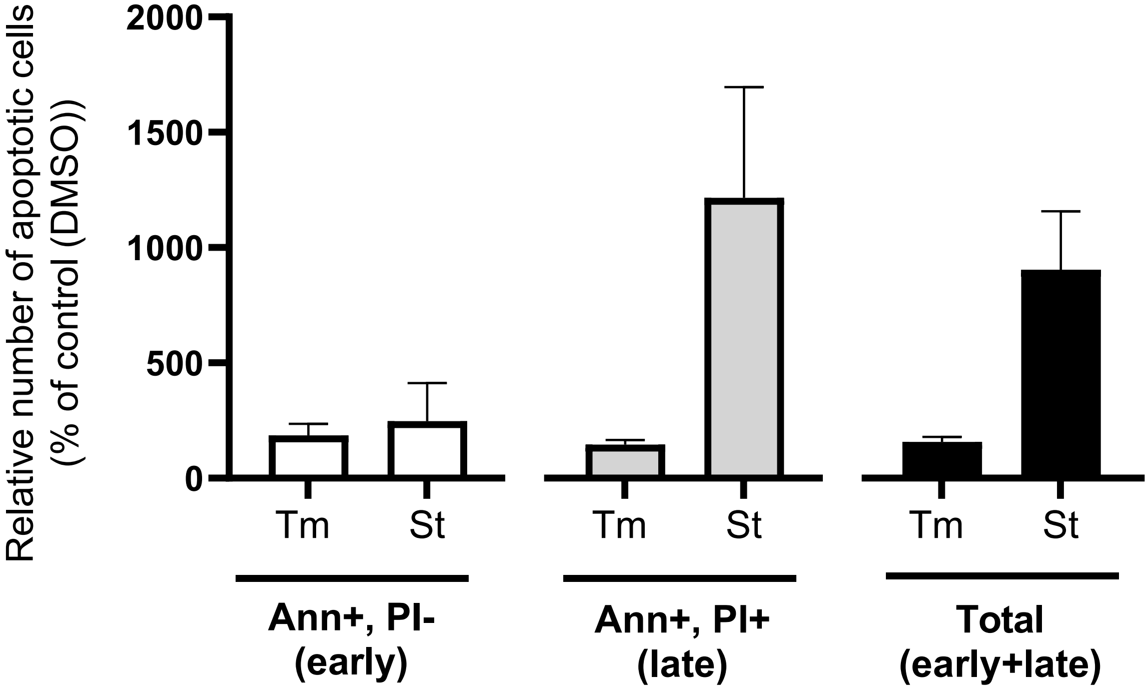

Supplementary data 19. Flow Cytometry Controls for Annexin V and PI Staining. BeWo cells were treated with Tm (3.5 µg/mL) a positive control for ER stress of St (1 mM) a positive control for apoptosis under normoxia (8% O_2) conditions during 24-h and 1-h respectively. A: Number of apoptotic cells was determined by FACS using Annexin V and PI staining. Number of early or late apoptotic cells are expressed as a percentage of the control DMSO. Ann: annexin V; DMSO: dimethylsulfoxyde; Early: early apoptosis; Late: late apoptosis; Mel: melatonin; PI: propidium iodide; St: staurosporine; Tm: tunicamycin; Total: total apoptosis. Data are shown as mean ± SD and were analyzed using an unpaired t-test (DMSO vs. Mel, *$p < 0.05$), n=7.

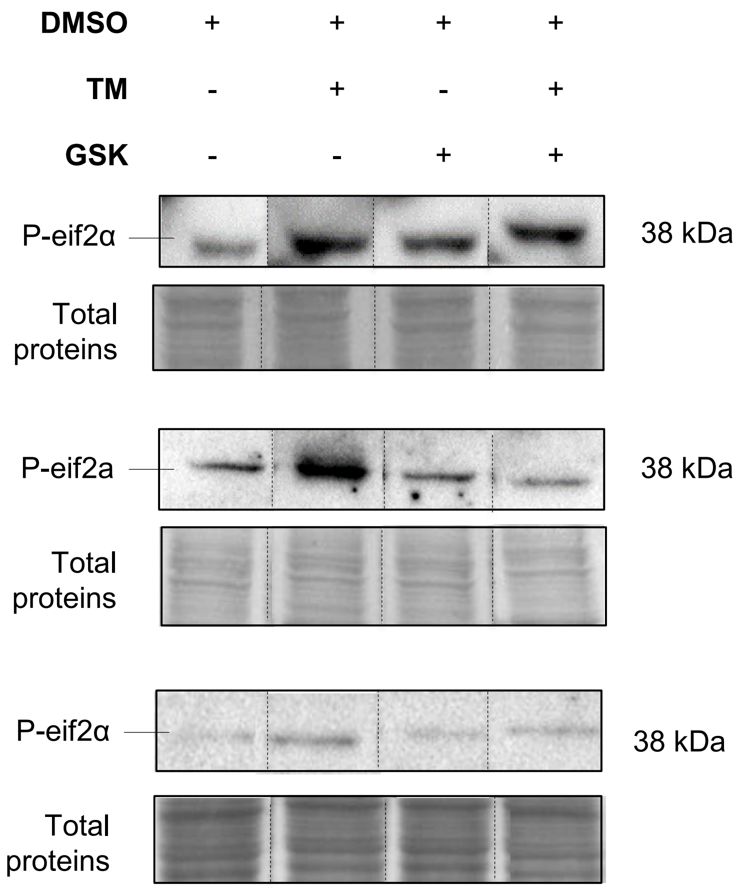

Supplementary data 20. A 24-h pre-treatment of GSK256157 (100 nM) failed to inhibit activation of p-eIF2α by TM in BeWo cells. DMSO: dimethylsulfoxyde; TM: Tunicamycin. To facilitate readers' experiences, the band order has been changed. Those modifications have been identified with dotted lines.

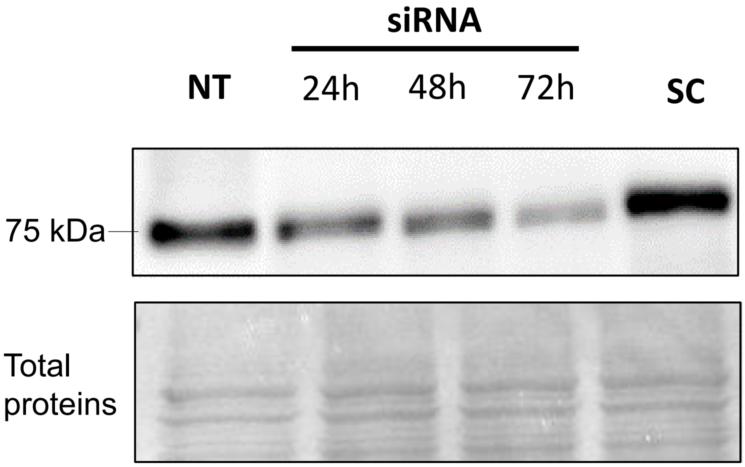

Supplementary data 21. Knockdown of PERK with specific siRNA is efficient at 72-h. NT: no treatment; SC: scramble.

PERK (siRNA PERK)

A

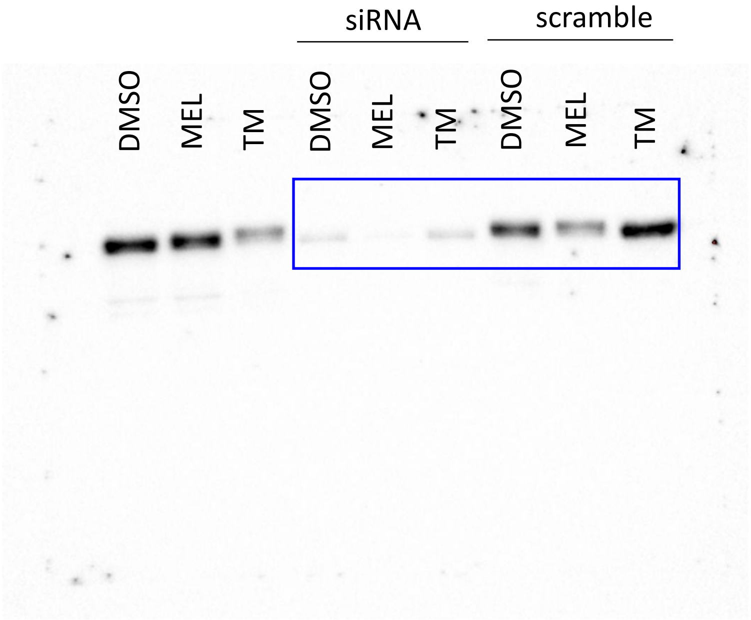

B

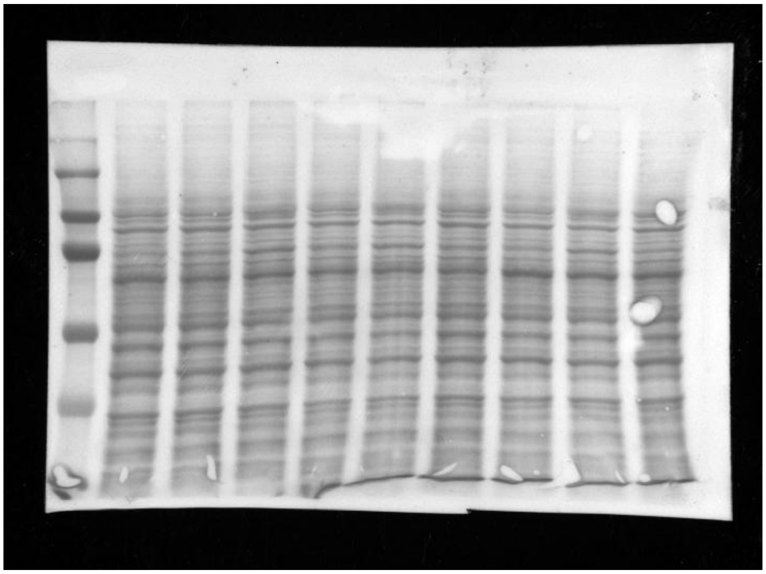

Supplementary data 22. This is the original blot of Fig. 6A. BeWo cells subjected to scramble (SC) or siRNA against PERK for 72-h were treated with the vehicle (DMSO 0.1%) or melatonin (1 mM) or TM (3.5 µg/mL) under normoxia (8% O_2) conditions during 24-h. A: PERK protein level was determined by Western blot. Equal protein amounts of cell lysates (35 µg) were subjected to Western blot assay using anti-PERK. Blue boxes mark the bands shown in Fig. 6A. B: Total protein was used for normalization. DMSO: dimethylsulfoxyde; Mel: melatonin; PERK: protein kinase R (PKR)-like; Tm: tunicamycin.

GRP78 (siRNA PERK)

A

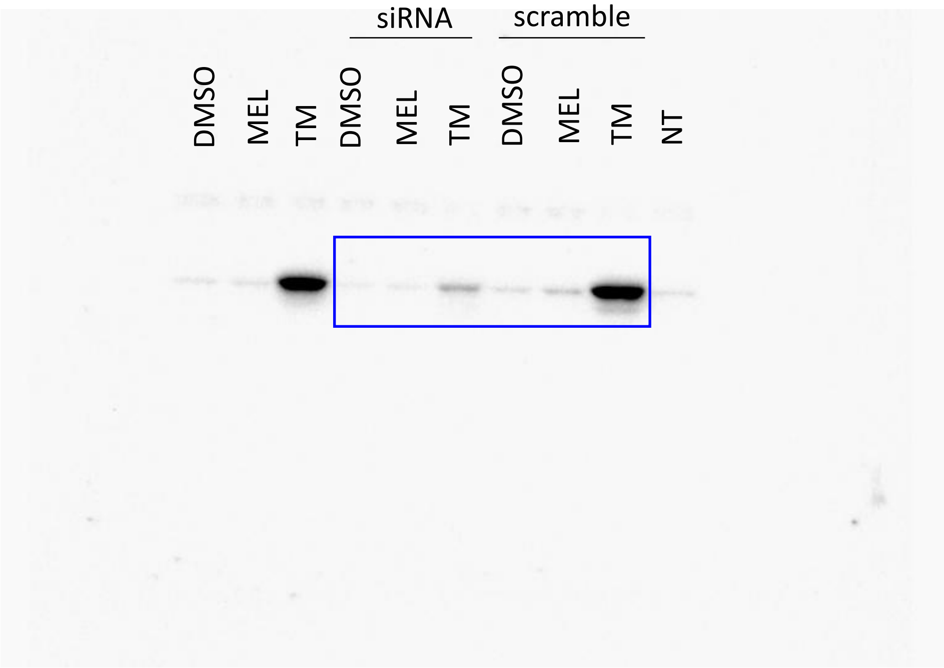

B

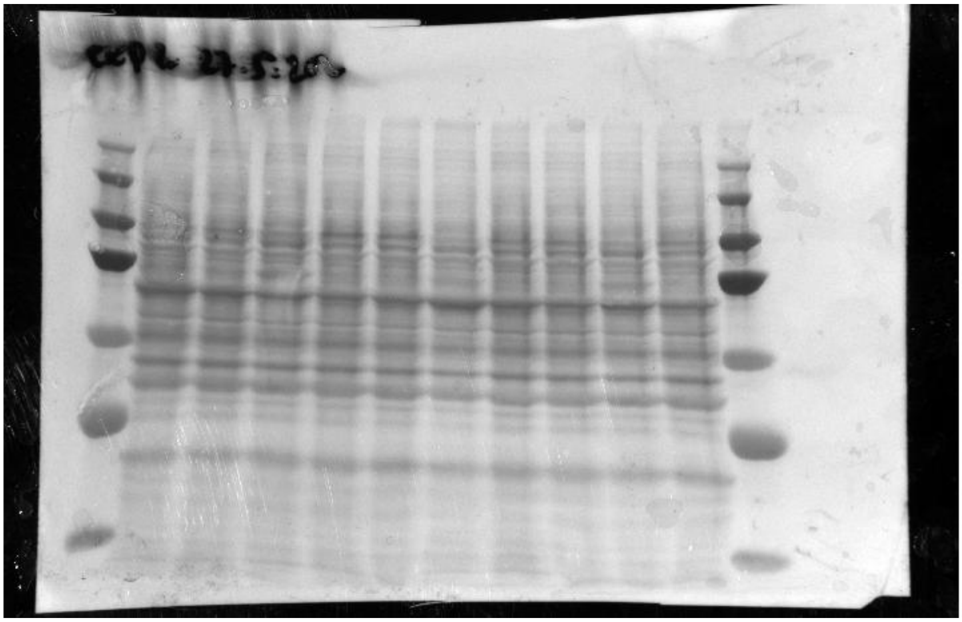

Supplementary data 23. This is the original blot of Fig. 6B. BeWo cells subjected to scramble (SC) or siRNA against PERK for 72-h were treated with the vehicle (DMSO 0.1%) or melatonin (1 mM) or TM (3.5 µg/mL) under normoxia (8% O_2) conditions during 24-h. A: GRP78 protein level was determined by Western blot. Equal protein amounts of cell lysates (35 µg) were subjected to Western blot assay using anti-GRP78. Blue boxes mark the bands shown in Fig. 6B. B: Total protein was used for normalization. DMSO: dimethylsulfoxyde; GRP78: Glucose-Regulated Protein 78; Mel: melatonin; Tm: tunicamycin.

P-eIF2α (siRNA PERK)

A

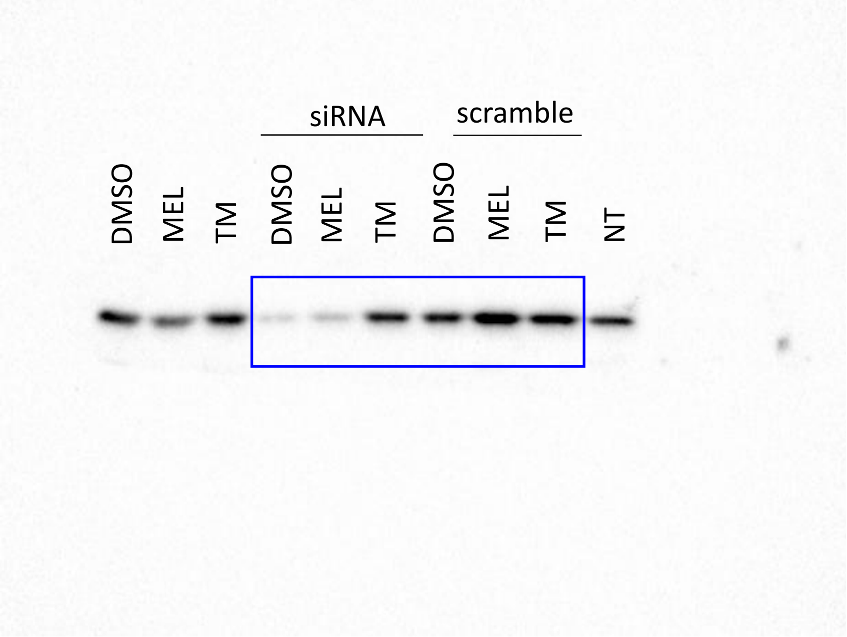

B

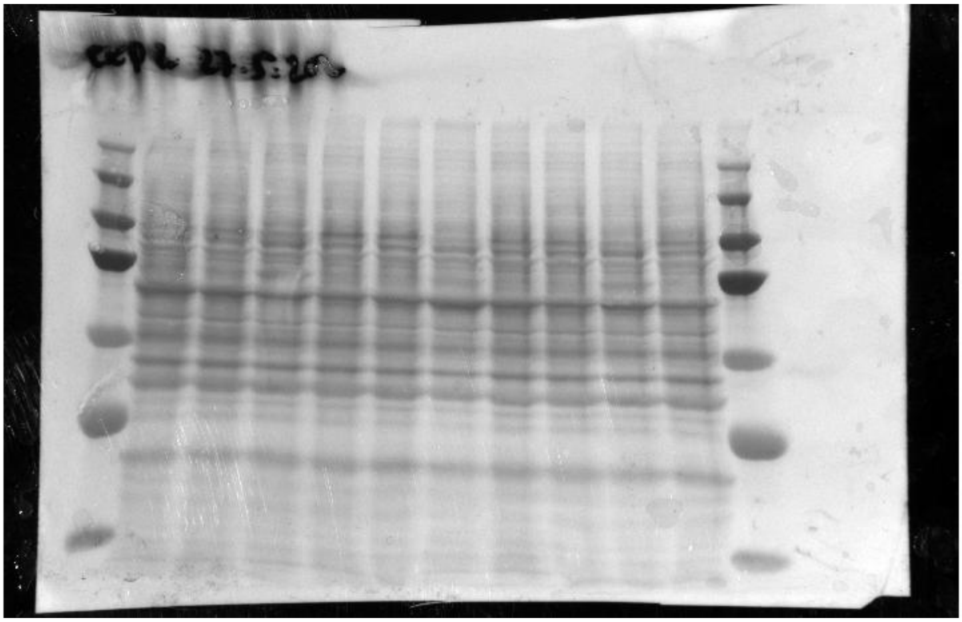

Supplementary data 24. This is the original p-eIF2α blot that was presented in Fig. 6C and D. BeWo cells subjected to scramble (SC) or siRNA against PERK for 72-h were treated with the vehicle (DMSO 0.1%) or melatonin (1 mM) or TM (3.5 µg/mL) under normoxia (8% O_2) conditions during 24-h. A: P-eIF2α protein level was determined by Western blot. Equal protein amounts of cell lysates (35 µg) were subjected to Western blot assay using anti-p-eIF2α. Blue boxes mark the bands shown in Fig. 6C and D. B: Total protein was used for normalization. DMSO: dimethylsulfoxyde; Mel: melatonin; P-eIF2α: Phosphorylation of the eukaryotic initiation factor 2 subunit alpha; Tm: tunicamycin.

eiF2α (siRNA PERK)

A

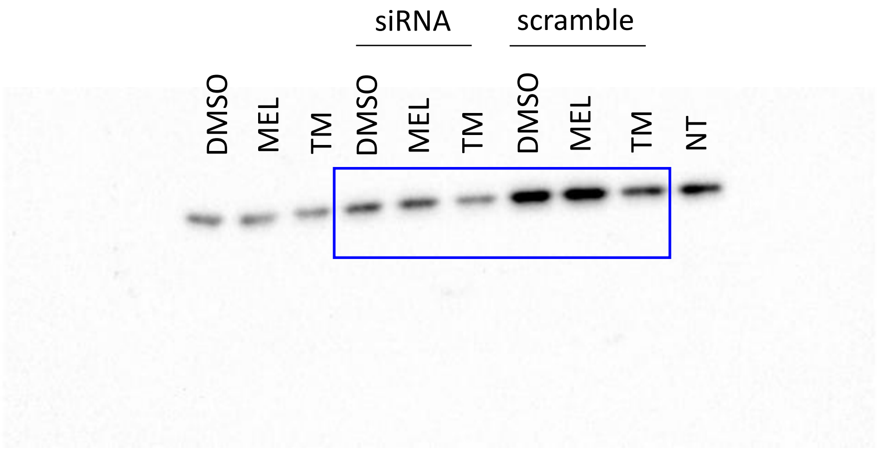

B

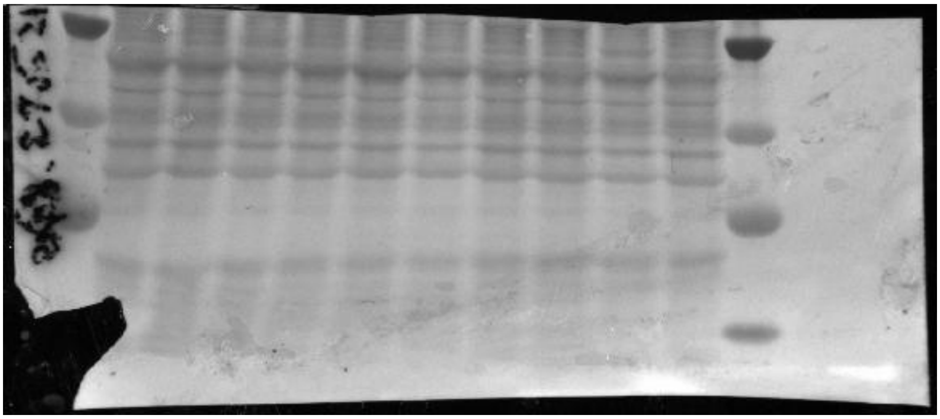

Supplementary data 25. This is the original p-eIF2α blot that was presented in Fig. 6C and E. BeWo cells subjected to scramble (SC) or siRNA against PERK for 72-h were treated with the vehicle (DMSO 0.1%) or melatonin (1 mM) or TM (3.5 µg/mL) under normoxia (8% O_2) conditions during 24-h. A: eIF2α protein level was determined by Western blot. Equal protein amounts of cell lysates (35 µg) were subjected to Western blot assay using anti-eIF2α. Blue boxes mark the bands shown in Fig. 6C and E. B: Total protein was used for normalization. DMSO: dimethylsulfoxyde; Mel: melatonin; eIF2α: eukaryotic initiation factor 2 subunit alpha; Tm: tunicamycin.

ATF4 (siRNA PERK)

A

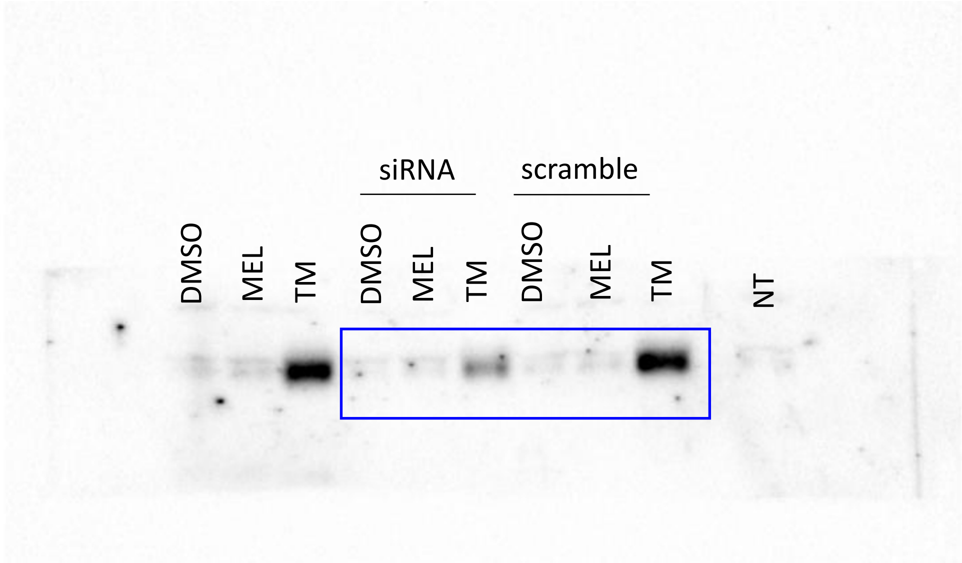

B

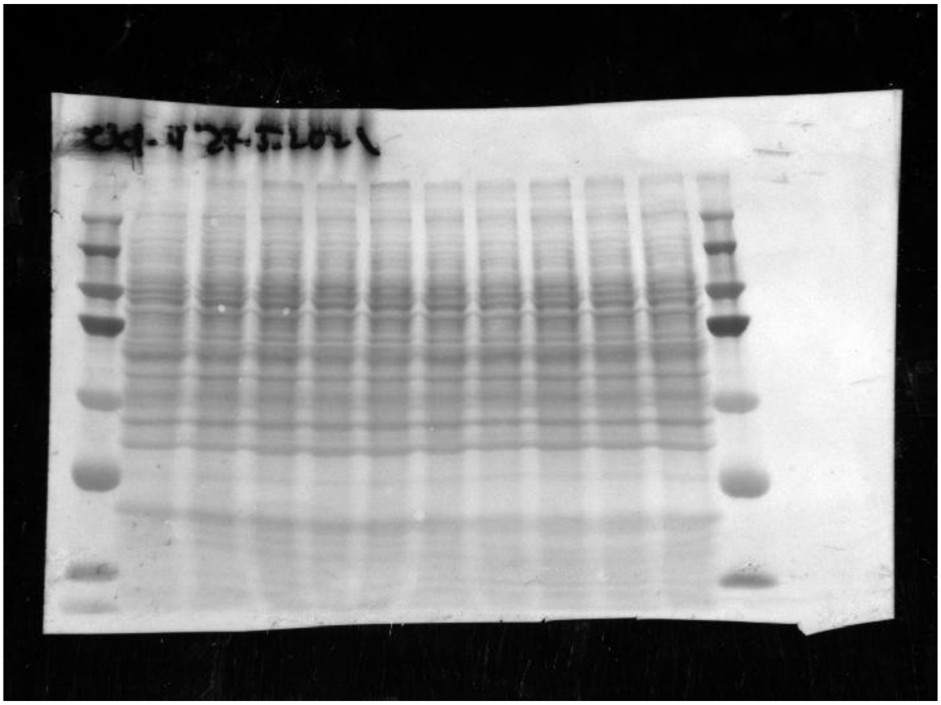

Supplementary data 26. This is the original p-eIF2α blot that was presented in Fig. 6F. BeWo cells subjected to scramble (SC) or siRNA against PERK for 72-h were treated with the vehicle (DMSO 0.1%) or melatonin (1 mM) or TM (3.5 µg/mL) under normoxia (8% O_2) conditions during 24-h. A: ATF4 protein level was determined by Western blot. Equal protein amounts of cell lysates (35 µg) were subjected to Western blot assay using anti-ATF4. Blue boxes mark the bands shown in Fig. 6F. B: Total protein was used for normalization. ATF4: Activating Transcription Factor 4; DMSO: dimethylsulfoxyde; Mel: melatonin; Tm: tunicamycin.
